# A human Caco-2-based co-culture model of the inflamed intestinal mucosa for particle toxicity studies

**DOI:** 10.1007/s44164-023-00047-y

**Published:** 2023-03-24

**Authors:** Maxi B. Paul, Marén Schlief, Hannes Daher, Albert Braeuning, Holger Sieg, Linda Böhmert

**Affiliations:** Department of Food Safety, German Federal Institute for Risk Assessment, Max-Dohrn-Str. 8-10, 10589 Berlin, Germany

**Keywords:** Intestinal immune system, In vitro model, Caco-2, Microplastics, Particle requirements suitable

## Abstract

The intestinal barrier is a complex interface of the human body, possessing the largest contact surface to nutrients and antigens and containing a major part of the immune system. It has to deal with continuous exposure to a broad mixture of essential, harmful, or useless substances and particles. In the context of plastic pollution and the ubiquitous occurrence of micro- and nanoplastics, oral exposure to such particles is of particular interest. Standard intestinal in vitro models, however, are unable to mimic the role of the immune system in the particle-exposed intestine. To allow for a closer look on the effect of particles on the intestinal immune system, we here developed a co-culture model to enable investigation of the epithelial brush border monolayer in a healthy and inflamed state. The model is based on well-established Caco-2 intestinal epithelial cells cultured in a Transwell™ system. Intraepithelial immune cells were mimicked by THP-1-derived M0-macrophages and MUTZ-3-derived dendritic cells. To fulfill the requirements needed for the investigation of particles, the co-culture system was developed without an additional matrix layer. Cell–cell contacts were established between interstitial and immune cells, and the Caco-2 standard cell culture medium was used, which is well-characterized for its role in defining the identity of particle dispersions. The model was characterized using confocal microscopy, membrane integrity measurements, and cytokine release assays from inflamed and healthy cells. Finally, the new co-culture model was used for investigation on polylactic acid, melamine formaldehyde resin, and polymethylmethacrylate plastic micro- and nanoparticles.

**Supplementary Information:**

The online version contains supplementary material available at 10.1007/s44164-023-00047-y.

## Introduction

The biggest and most complex part of the human immune system is located in the intestine, which is also the largest surface of our body that is constantly exposed to nutrients and xenobiotic substances [1]. Therefore, its homeostasis and balance are vital to its function. Perturbation can cause severe effects such as chronic inflammation, disrupted epithelial barrier function, and tissue damage [2]. The intestinal barrier, in general, consists mainly of intestinal epithelial cells (enterocytes), M-cells, and mucus-producing goblet cells, as well as of epithelium-associated immune cells, such as macrophages, dendritic cells, and B-cells [3, 4]. Tight junctions enable a strong connection between enterocytes to form a semi-permeable and selective mucosal barrier. Through different uptake mechanisms, antigens may pass this barrier to be taken up mainly by macrophages and dendritic cells, which are located in close vicinity to the epithelial cells [1]. After activation, these immune cells produce cytokines and induce immune responses and phagocytosis [1]. So-called M0-macrophages are capable of taking up antigens prior to their passage of the intestinal barrier. After activation through antigens, M0-macrophages polarize either into pro-inflammatory M1-macrophages or into anti-inflammatory M2-macrophages to regulate the immune response [5]. Lipopolysaccharide (LPS), for example, is a stimulus known to induce polarization into M1-macrophages. Additionally, M-cells and goblet cells are also responsible for antigen uptake.

Due to an increasing toxicological focus on the oral uptake of compounds and particles that might interact with the intestinal immune system, easy-to-use in vitro models are needed. This is especially true when it comes to nano- and microparticles, where particularly plastic pollution, the use of intentionally produced small plastic particles, and especially the degradation of larger plastics to such small particles have raised concerns about potential human health effects in the past years. Due to the fact that plastic materials are a very diverse group of different polymers with a huge variety of physicochemical characteristics, risk assessment is challenging. It is known that nano- and microplastics may be taken up via the intestinal barrier [6–10]. Based on these data, the European Food Safety Agency (EFSA) has concluded that particles sized below 150 µm are able to cross the gastrointestinal barrier in general, whereas 10 µm is suggested as the upper limit for cellular uptake. The potential systemic bioavailability of plastic particles is limited to those smaller than 1.5 µm [11]. The impact of different plastic particles on enterocyte models has already been investigated and revealed material-, size-, and surface-dependent effects related to inflammation or oxidative stress [7, 12, 13]. Nonetheless, effects of micro- and nanoplastics on intraepithelial immune cells remain largely unknown.

When it comes to experiments with nano- or microparticles, there is a specific need to use the same culture medium composition for incubation and dispersion of particles for all models whose results shall be compared. This is important because the composition of cell culture media has a major influence on the stability of the particle dispersion and therefore the dosimetry, and also on the composition of the protein corona which modulates a particle’s appearance and identity [14–16].

Since a few decades, in vitro models have been developed, used, and adapted to answer different research questions dealing with the intestinal barrier. A well-suited cell line mimicking the most abundant intestinal cell type, the enterocyte, has been established in the 1970s [17]. This carcinoma cell line, named Caco-2, forms a cell monolayer and spontaneously differentiates to a brush border appearance, with tight junctions and polarized cells with microvilli on the apical side [18, 19]. Cultured on permeable Transwell™ membranes, it is suitable for transport studies, for which access to both basolateral and apical compartments is necessary [20–22]. The basic Caco-2 model further evolved by introduction of other cell types to mimic different features of the in vivo situation. The well-established co-culture of differentiated Caco-2 cells and HT29-MTX, which represent mucus-producing cells inside the intestinal monolayer, was used to demonstrate enhanced uptake and transport of nano- and microplastics compared to the monoculture [7, 8, 23]. To focus more on the impact of immune cells, Leonard et al. developed a model consisting of Caco-2 cells, dendritic cells, and macrophages. Primary immune cells were obtained from blood samples and embedded in a collagen type I gel on a Transwell™ insert [24]. Caco-2 cells were seeded on top and the model was co-cultivated for 21 days. The model was inflamed using LPS, and cytokine release was quantified. The usage of primary cells is thought to closely reflect the in vivo situation in humans, but especially high costs, cultivation time, and the variance of donor characteristics restrict their applicability and the reproducibility of the entire model. Additionally, the matrix between the immune cells limits their movement and mutual cell–cell contacts. The introduction of cell line-based immune cells in the Caco-2 system started in 2001 by co-culturing undifferentiated THP-1 cells with differentiated Caco-2 cells to investigate phagocytic activities of macrophages. However, the THP-1 cells were used as proliferating cells, which mainly express characteristics of monocytes, but not of macrophages [25]. Progress was achieved in 2010 by co-culturing PMA-differentiated M0-macrophages from THP-1 with differentiated Caco-2 cells. It was shown that macrophages can increase the uptake of microparticles into intestinal cells [26]. Nevertheless, both approaches lacked cell–cell contacts and did not investigate inflammatory responses. In 2016, PMA-differentiated THP-1 cells and undifferentiated MUTZ-3 cells were embedded in type I collagen on the apical side of Transwell™ membranes and Caco-2 cells were seeded on top of the layer [27]. MUTZ-3 cells are derived from a patient with acute myelomonocytic leukemia and represent dendritic cells when differentiated. The above setup enabled again only limited cell–cell contact and movement, which might be also problematic when studying the transport of particles. Additionally, co-culturing time was very long, as the immune cells had to be co-cultivated for the entire 3 weeks of Caco-2 differentiation, and possible changes in differentiation of the immune cells during this time were not monitored. To overcome the problem of the long co-cultivation and matrix limitations, another in vitro model in 2017 combined differentiated Caco-2 cells and PMA-differentiated THP-1 by seeding macrophages on the bottom of a well and mounting the Transwell™ insert containing the intestinal monolayer above, without enabling cell–cell contact [28]. This permits an exchange of secreted factors like cytokines, but still does not allow for cell–cell contacts for intraepithelial immune cells, as present in vivo*.* The cells were additionally primed and stimulated before co-culturing to induce controlled inflammation, which was monitored by cytokine and lactate dehydrogenase release. In this model, the co-culture time was short, but two different cell culture media were used.

None of the above described previous co-culture models fully reached the requirements for a model of the intestinal epithelium with intraepithelial immune cells, suitable to study small particles like nano- and microplastics. Therefore, the goal of this work was to establish in vitro co-culture models of the healthy and inflamed intestinal mucosa that fulfill the following criteria:

- The model should mimic the in vivo situation of intraepithelial immune cells in the mucosa in their normal and inflamed status.
- To ensure reproducibility across different laboratories and comparability, human permanent cell lines should be used.
- The in vitro model should be suitable for investigation of substances and for particles like nano- and microparticles of different materials, e.g., plastic polymers. Therefore, no additional biopolymer layer should be introduced into the system, as this may have an influence on the transport of particles.
- The co-culture model should be maintained in the same cell culture medium as the monoculture, to ensure comparability of results for particles, because the medium composition has a major influence on their dispersion stability, dosimetry, protein corona, and therefore particle appearance and identity.
- Immune cells should have direct cell–cell contact to the Caco-2 cells to enable cellular interaction; they are therefore to be seeded on the basolateral side of the Transwell™ membrane.
- Co-culture time should be as short as possible to maintain properties of the immune cells.
- Activation of immune cells in the model by an inflammatory stimulus should be possible to investigate the effect of inflammation on the response to the presence of particles.

Based on these specifications, we developed in vitro models of the healthy and inflamed intestine by co-culturing cell-line derived enterocyte-like cells (Caco-2 cells) with macrophages (THP-1) and dendritic cells (MUTZ-3) on the basolateral side of intestinal cells.

## Methods/experimental

### Chemicals

If not stated otherwise, all chemicals were purchased from Merck KgaA (Darmstadt, Germany), Carl Roth GmbH & Co. KG (Karlsruhe, Germany), or Sigma-Aldrich Chemie GmbH (Taufkirchen, Germany). The cytokines were ordered from ImmunoTools GmbH (Friesoythe, Germany).

### Plastic particles

Polylactic acid micro- and submicroparticles with a mean diameter of 2000 nm (PLA2000, stock conc.: 2.40 × 10^9^ particles/mL) and 250 nm (PLA250, stock conc.: 1.2 × 10^12^ particles/mL) were purchased from Micromod Particle Technology GmbH (Rostock, Germany) with a fluorescence label of Ex/Em 497/530 nm (PLA-greenF, prod.-no: 51–00–252 and 51–00–203). Melamine formaldehyde resin submicroparticles with a mean diameter of 366 nm (MF366, stock conc.: 3.07 × 10^10^ particles/mL) were purchased from Microparticles GmbH (Berlin, Germany) and have a fluorescence label of Ex/Em 560/584 nm (charge: MF-FluoOrange-S886–1). Polymethylmethacrylate nanoparticles with a mean diameter of 25 nm (PMMA25, stock conc.: 1.20 × 10^15^ particles/mL) were ordered from Creative Diagnostics (New York, USA) with a fluorescence label of Ex/Em 475/510 nm (DiagPoly™ Plain Fluorescent PMMA nanoparticles, cat.-no.: DNG-P010). All particles were characterized in a previous publication [29]. In short, these particles represent different materials with food relevance: PLA2000 was used as a representative for polydisperse particles from the micro- to the nanometer range. PLA250 and MF366 are particle species of different material and hydrophobicity in the submicrometer range. The PMMA25 particles are a surrogate for nanoplastics. Dynamic light scattering in water as well as in culture medium showed no differences in the hydrodynamic diameter of the particles between the two media. For experiments, the unit was converted to particle surface per milliliter because it ensures comparability between particles of different sizes since the surface of particles increases with decreasing size.

### Cell cultivation and maintenance

The human intestinal cell line Caco-2 (ECAC: 86,010,202) was obtained from the European Collection of Authenticated Cell Cultures (Salisbury, UK). Human monocytic THP-1 cells (DSMZ-No: ACC 16) and the human myelomonocytic leukemia cell line MUTZ-3 (DSMZ-No: ACC 295) were purchased from the Leibniz Institute DSMZ-German Collection of Microorganisms and Cell Cultures GmbH (Braunschweig, Germany). All cells were maintained at 37 °C and 5% CO_2_. Caco-2 cells were cultivated in Dulbecco’s modified Eagle medium (DMEM, Pan-Biotech GmbH, Aidenbach, Germany) supplemented with 10% fetal calf serum superior (FCS superior), 10^5 ^units/L penicillin, and 100 µg/mL streptomycin (1% P/S; Capricorn Scientific GmbH, Ebsdorfergrund, Germany) in T75 flasks. Cells were sub-cultured as soon as they reach 80 to 90% confluence (every 2 to 3 days). For passaging, cell culture medium was aspirated and cells washed with phosphate-buffered saline (PBS). Accordingly, cells were incubated with 0.05% trypsin-ethylenediaminetetraacetic acid (EDTA) at 37 °C for 5 min. The reaction was stopped by adding 8.5 mL cell culture medium. The cell suspension was harvested and cells centrifuged at 300 × *g* for 5 min. The cell pellet was resuspended in fresh cell culture medium and split into new T75 flasks. The THP-1 cell line was cultivated in T75 flasks in Roswell Park Memorial Institute 1640 (RPMI; Pan-Biotech GmbH, Aidenbach, Germany) medium, which was supplemented with 10% FCS superior and 1% P/S. These cells were passaged every 3 to 4 days by harvesting a part of the cells and convey them into a new flask with fresh culture medium. The human bladder carcinoma cell line 5637 (ATCC HTB-9) that was needed for MUTZ-3 cultivation was maintained in RPMI 1640 supplemented with 10% FCS superior and 1% P/S in T150 flasks at concentrations between 3 and 4 × 10^6^ cells/mL in a 40-mL culture medium. The dissipated cell culture medium was collected every 2 to 3 days, filtered, and stored at − 20 °C for further usage. MUTZ-3 cells were grown in 24-well plates filled with 2 mL/well Alpha-modified minimum essential medium Eagle (α-MEM Eagle; Pan-Biotech GmbH, Aidenbach, Germany) supplemented with 20% FCS superior, 20% conditioned medium of 5637 cells, 1% P/S, and 1% l-glutamine (PAN-Biotech GmbH, Aidenbach, Germany). This cell line needs to be maintained at concentrations between 1 and 2 million cells/mL and was therefore sub-cultured 1:2 every 4 to 5 days. Cells were gently resuspended in each well and half of the cell suspension poured in a new well. Every well was filled with 1 mL of fresh cell culture medium. Table 1 gives an overview of the different media used and for what purposes:

**Table 1:** Overview of applied media for different purposes

Abbreviation	Medium composition	Application field		
		Proliferating cells	Differentiating cells	Co-cultivation and particle characterization
DMEM +	DMEM With 4.5 g/L glucose With L-glutamine With sodium pyruvate With 3.7 g/L NaHCO_3_ + 1% P/S + 10% FCS superior	Caco-2	Caco-2	X
RPMI +	RPMI 1640 With l-glutamine With 2.0 g/L NaHCO_3_ + 1% P/S + 10% FCS superior	THP-1 and 5637	THP-1	
MEM +	α-MEM Eagle With ribonucleosides With deoxyribonucleosides With 2.2 g/L NaHCO_3_ + 1% P/S + 20% FCS superior + 20% supernatant of 5637 + 1% l-glutamine	MUTZ-3		
Adaption medium	MEM + and DMEM + in different percentages		MUTZ-3	

### Differentiation of cells

Caco-2 cells, used as intestinal epithelial cells for the co-culture, were treated as described in a protocol by Stock et al. [8]. In short, 12-well Transwell™ plates (Corning Incorporated, New York, USA) with polycarbonate membrane inserts that exhibit a 1.12-cm^2^ growth area and 3-µm pore size are used. Cells were seeded on the membrane of the Transwell™ inserts at a density of 50,000 cells/membrane and maintained in DMEM + . As soon as the cells reached confluence, they differentiated without additional treatment into a functional intestinal-like monolayer within 3 weeks. This differentiation is well known and has been characterized by several groups [30, 31]. Besides, the differentiation of Caco-2 cells was characterized in our group with respect to enzyme expression, xenobiotic metabolism, proteomic pattern, cell cycle arrest, and morphology, and the model has been used in our group for more than a decade [15, 22, 32, 33]. Morphological characteristics of differentiated Caco-2 cells are shown in Figure S4 in the supporting information. The cell culture medium was changed every 2 to 3 days.

Differentiation of THP-1 cells into M0-macrophages followed the protocol from Lund et al. (2016) [34]. In short, cells were seeded at a density of 5 × 10^5^ cells/mL and let differentiate for 3 days. The differentiation was initiated by adding 25 nmol/L Phorbol 12-myristate 13-acetate (PMA).

The differentiation of MUTZ-3 cells to dendritic-like cells is not as standardized as the differentiation of THP-1 or Caco-2 cells. Therefore, prior to investigating the behavior of MUTZ-3-derived dendritic cells under co-culture conditions, the most suitable differentiation protocol of MUTZ-3 into immature dendritic cells had to be chosen. This was done by testing the following differentiation protocols adapted from Nyambura et al. [35]. According to this protocol, 1 × 10^5^ cells/mL was seeded in a 24-well plate and incubated with differentiation medium in a final volume of 2 mL. The basic medium condition consisted of α-MEM Eagle + 20% FCS superior + 1% P/S + 1% l-glutamine and MUTZ-3 cells were differentiated with one of the following supplements:

1. 10% conditioned medium of 5637 cells
2. 10% conditioned medium of 5637 cells, 20 ng/mL interleukin 4 (IL-4), 100 ng/mL granulocyte–macrophage colony-stimulating factor (GM-CSF)
3. 20% conditioned medium of 5637 cells, 20 ng/mL IL-4
4. 20% conditioned medium of 5637 cells, 20 ng/mL IL-4, 100 ng/mL GM-CSF

The cells were differentiated for 7 days. Medium change was performed after 4 days by removing 1 mL of the differentiation medium and adding half amounts of cytokines or conditioned medium (depending on the condition) in 1 mL of fresh cell culture medium. Cells maintained in the usual MEM + served as control. After 7 days, cells were harvested and used for real-time quantitative polymerase chain reaction (RT qPCR) to examine differentiation markers. Additionally, the morphology of the cells after differentiation was investigated with the inverse microscope Axio Observer d1 (Carl Zeiss, Oberkochen, Germany). The brightfield channel and × 50 magnification were used.

### Co-culture establishment

**Fig. 1:**
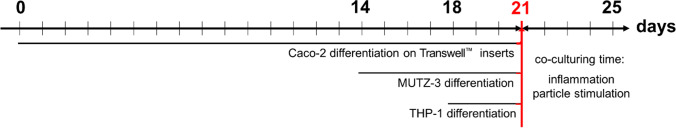
Timeline of the co-cultivation procedure. Caco-2 cells were differentiated on Transwell™ inserts for 21 days. MUTZ-3 and THP-1 cells were differentiated in parallel in different cell cultivation dishes starting on days 14 or 18, respectively. Immune cells were added on the basolateral side of the membrane on day 21 (cp. red line in the figure). During the subsequent co-culture time, cells were treated with LPS for inflammation and subsequently incubated with nano-, submicro-, or microparticles

In general, the co-culture setup should comprise the following features: Caco-2, THP-1, and MUTZ-3 cells are differentiated in monoculture into intestinal cells, M0-macrophages, and dendritic cells, respectively. Afterwards, cells are co-cultured for 5 days in 12-well Transwell™ plates and maintained in DMEM + . During these days, immune cells should interact with the intestinal barrier. Additionally, the model can be inflamed with an inflammatory trigger, and can be incubated with micro-, submicro-, and nanoparticle dispersions. As an inflammatory stimulus, the bacterial endotoxin LPS was added to the cells for 24 h. Thereof, two models arose: a healthy co-culture model to investigate the intestinal immune system under balanced conditions, and an inflamed co-culture model to examine changes in a stressed environment. Figure 1 gives an overview on the envisaged timeline of the co-culture model.

As described in the “Introduction,” the immune cells should reach the following criteria: They should (i) express properties of immature dendritic cells or M0-macrophages, (ii) interact with the Caco-2 monolayer, and (iii) tolerate the DMEM + medium used for co-culturing. To test for these criteria, a stepwise approach was chosen: We started with a comparison of differentiation protocols for the immune cells. The second step was to verify the survival of the cells and maintenance of their differentiation marker in DMEM + . This was followed by a simulation experiment with THP-1 and MUTZ-3 cells, consisting of all steps that are needed for the co-culture model, but without introducing Caco-2 cells. Differentiation and inflammatory markers were checked via RT qPCR. Finally, cells were combined in the co-culture and investigated for their properties. To this end, cells were stained individually and morphological changes monitored by using confocal microscopy. Before embedding the membrane for microscopy, the model was treated with LPS (inflamed model) or not (healthy model), and incubated with particles for 24 h each.

#### Introducing THP-1-derived M0-macrophages into the co-culture model

**Fig. 2:**
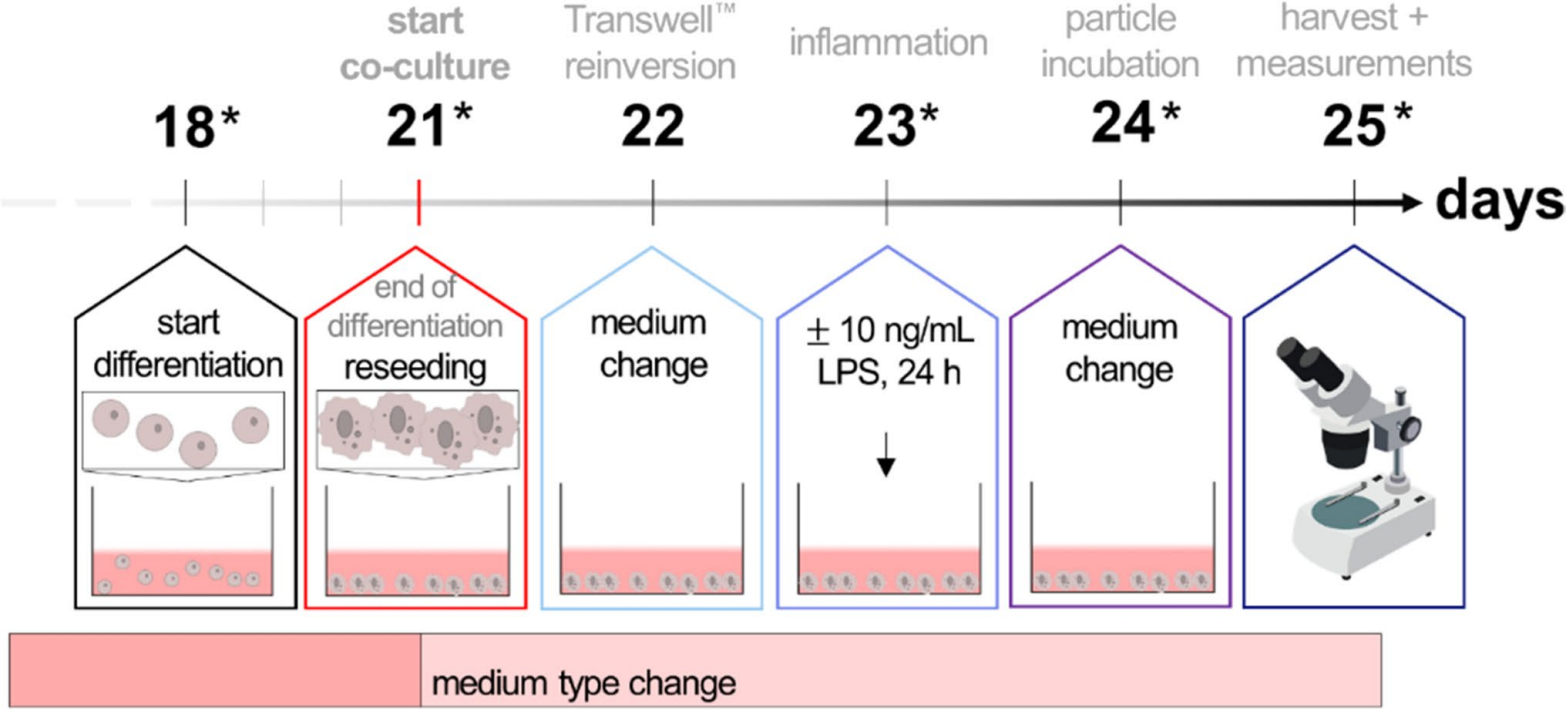
Timeline for the differentiation and adaption of THP-1-derived M0-macrophages. The number of days goes by the complete co-culture timeline as given in Fig. 4. The cells were differentiated with 25 nM Phorbol 12-myristate 13-acetate (PMA) for 72 h in RPMI + and co-culture was simulated in DMEM + . The simulation with undifferentiated cells and differentiated cells in RPMI + served as controls. The cells were reseeded on day 21, received a medium change on days 22 and 24, and were inflamed with either 10 ng/mL LPS (inflamed model) or maintained with culture medium (healthy model) for 24 h on day 23. Stars indicate days on which cells were analyzed via fluorescence microscopy and harvested for RT qPCR

To incorporate M0-macrophages into the co-culture system, two different aspects needed to be considered: (i) PMA-driven differentiation of THP-1 cells into M0-macrophages and (ii) the possibility of verifying the behavior of these cells under co-culture conditions. Therefore, the medium of the THP-1 cells needed to be changed to the intestinal co-culture medium. Two aspects needed to be considered for the adaption of THP-1-derived M0-macrophages to the required co-cultivation conditions, namely whether these M0-macrophages maintain their differentiated state (1) in the required co-culture medium (DMEM supplemented with 10% FCS superior and 1% P/S), and (2) during the time of co-culturing until the end of incubation? Both aspects were addressed with an experimental setup that mimicked, in monoculture, the envisaged whole co-culture design, encompassing differentiation, combination of cell types, medium change, and inflammation, as well as the incubation time steps, with subsequent cell harvest for RT qPCR analysis (see the methods section, 2.8; Fig. 2). In detail, THP-1 cells were differentiated into M0-macrophages as described above. As surrogate for the combination of cell types to form the co-culture, cells were incubated with trypsin/EDTA for 7 min and the cell suspension collected in two different tubes and pelleted by centrifugation at 180 × *g* for 8 min. The cell pellets were resuspended either in RPMI 1640 (RPMI + , control) or DMEM (DMEM + , co-culture environment) and seeded in 12-well plates at a density of 5 × 10^5^ cells/mL. This represents day 21 of the co-cultivating procedure. Proliferating THP-1 cells cultivated in RPMI + served as undifferentiated control to examine basal gene expression. On the next day (corresponding to day 22 in the co-culture), a medium change was performed to mimic re-inversion of the Transwell™ inserts. The cells were then incubated either with 10 ng/mL LPS (equal to inflamed co-culture) or received a medium change (equal to healthy co-culture) on day 23 for 24 h. On day 24, another medium change was implemented to simulate the start of a potential incubation for additional 24 h. Finally, the simulation ended on day 25. The cells were harvested on days 18, 21, 23, 24, and 25 as indicated by asterisks in Fig. 2, and further processed for RT qPCR (see methods section, 2.8). To monitor the morphology of the THP-1 cells during the co-culture simulation, the inverse microscope Axio Observer d1 (Carl Zeiss, Oberkochen, Germany) in the brightfield channel and with × 50 magnification was used. Further analysis of THP-1-derived M0-macrophages in co-culture is described in the methods Section (2.5).

#### Introducing MUTZ-3-derived dendritic cells into the co-culture model

**Fig. 3:**
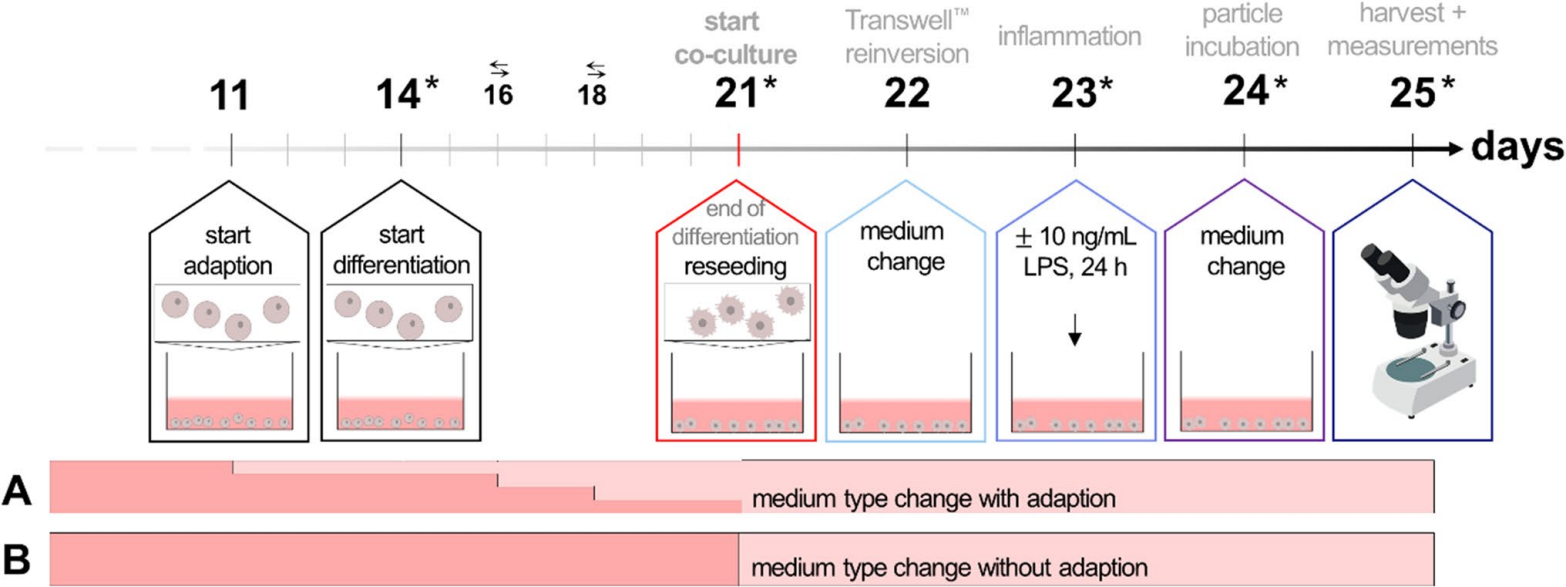
Timeline of the MUTZ-3 simulation experiment to mimic the co-culture steps in a monoculture. **A** Simulation experiment with medium adaption. The cells were differentiated with 20 ng/mL IL-4 and 100 ng/mL GM-CSF for 7 days in a gradual adaption from MUTZ-3 to DMEM + to accustom the cells to the surroundings during co-cultivation. Medium changes are indicated by opposing arrows. The co-culture procedure was simulated in DMEM + . The simulation with undifferentiated cells and differentiated cells in MEM + served as control. The cells were reseeded on day 21, received a medium change on days 22 and 24, and were inflamed with 10 ng/mL LPS for 24 h on day 23. As negative control, cells were incubated without LPS. Stars indicate days on which cells were additionally harvested for analysis. **B** Simulation experiment with medium type change from MUTZ-3 to DMEM + , but without adaption

After a suitable differentiation protocol for immature dendritic cells had been chosen, the adaption of these cells to the co-culture conditions was analyzed. These co-culture simulation experiments were performed similar to the experiments described for the THP-1-derived M0-macrophages in the methods Section (2.5), and additionally with either a stepwise gradual adaption to DMEM + before and during the differentiation (Fig. 3A), or without adaption (Fig. 3B). In short, the MUTZ-3 cell pellets were resuspended after differentiation either in MEM + (control) or in DMEM + (co-culture environment), or a stepwise change (25, 50, 75, and 100%) before and during the differentiation period from MEM + to DMEM + . The cells were seeded in 12-well plates at a density of 5 × 10^5^ cells/mL. This represents day 21 of the co-cultivating procedure. Proliferating MUTZ-3 cells cultivated in MEM + served as undifferentiated control and were used to examine basal gene expression. On the next day (corresponds to day 22 in the co-culture), a medium change was performed to mimic re-inversion of the Transwell™ inserts. The cells were then incubated either with 10 ng/mL LPS (equal to inflamed co-culture) or received a medium change (equal to healthy co-culture) on day 23. On day 24, another medium change was implemented to simulate the start of a potential incubation for 24 h. Finally, the simulation ended on day 25. The cells were harvested on days 18, 21, 23, 24, and 25 as indicated by asterisks in Fig. 3 and further processed for RT qPCR (see methods section, 2.8). To monitor the morphology of the MUTZ-3 cells during the co-culture simulation, the inverse microscope Axio Observer d1 (Carl Zeiss, Oberkochen, Germany) in the brightfield channel was used at × 50 magnification. Further analysis of MUTZ-3-derived immature dendritic cells in co-culture is described in the method Section (2.4).

**Fig. 4:**
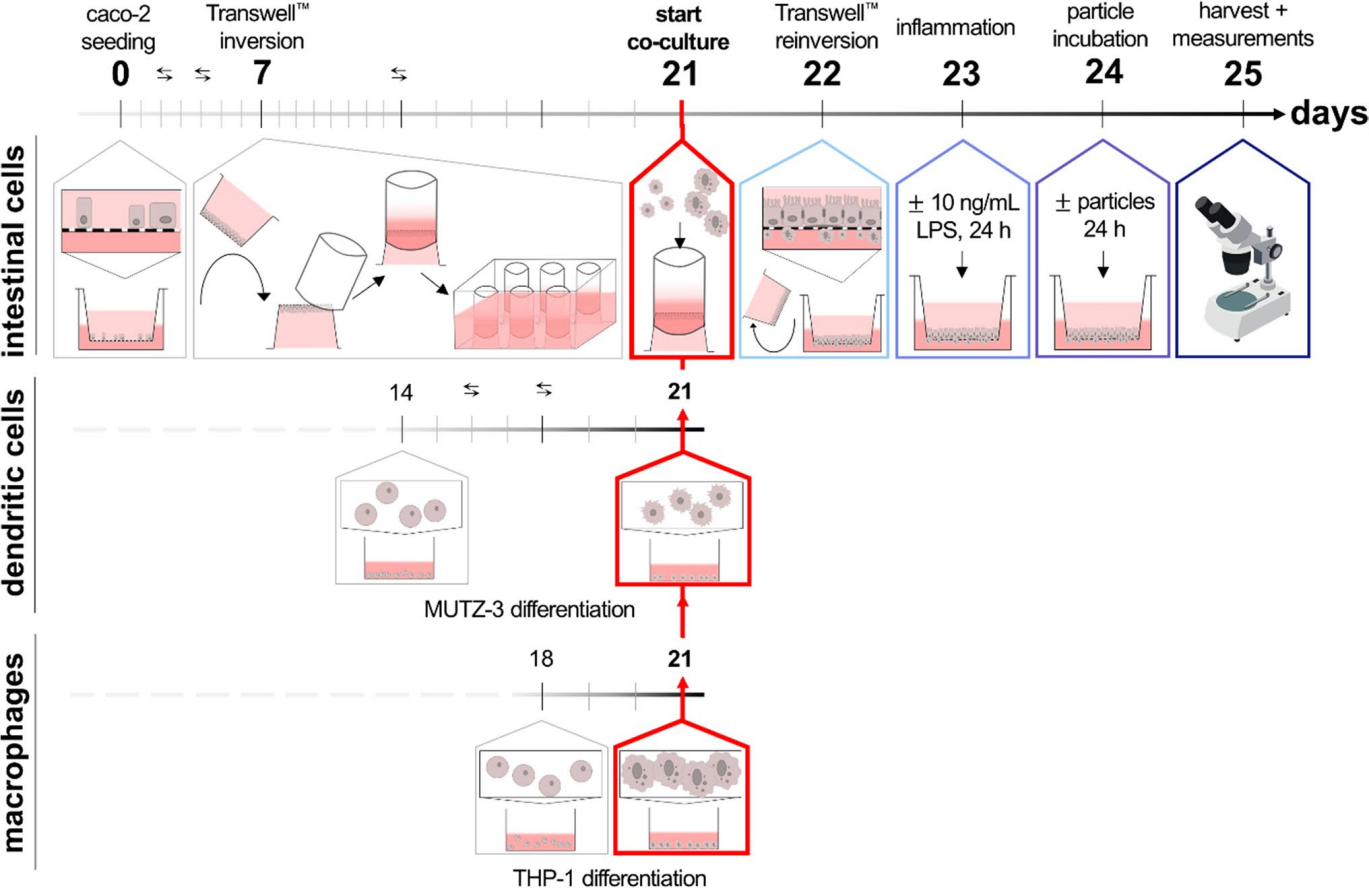
Timeline of the final co-culture procedure. Caco-2 cells were seeded on Transwell™ inserts on day 0 and let differentiate into intestinal cells for 21 days. The Transwell™ inserts were inverted on day 7. MUTZ-3 were differentiated to dendritic cells from days 14 to 21 with medium adaption, and THP-1 cells were differentiated to M0-macrophages from day 18 to day 21. The immune cells were added to Caco-2 cells on day 21 from the basolateral side of the Transwell™ membrane. The Transwell™ inserts were reinverted on day 22. The model received a medium change (healthy model) or was inflamed with 10 ng/mL LPS (inflamed model) for 24 h on day 23. Potential incubation with particles was ensued on day 24 for 24 h. The measurements and final harvest took place on day 25. Opposing arrows indicate medium changes

The timeline in Fig. 4 depicts the final procedure of the co-culture model. On day 0, Caco-2 cells were seeded for differentiation on Transwell™ inserts as described in the methods Section (2.3). On day 7, the Transwell™ inserts were inverted in modified and sterilized tip boxes, as previously described by Lichtenstein et al. [17, 18]: The boxes were filled with DMEM + beforehand. Silicone tubes were mounted carefully on the basolateral side of the inserts and placed in the medium-filled tip box. On day 14, 1.5 mL P/S was added to the tip box to prevent bacterial contamination. Additionally, the MUTZ-3 cells were differentiated for co-cultivation as described in the methods Section (2.5). The THP-1 cells were differentiated starting on day 18 as described in the methods Section (2.4). On day 21, the immune cells were harvested, stained (see methods section, 2.9), and resuspended in DMEM + . In total, 100,000 cells of each differentiated MUTZ-3 and THP-1 were added into the silicone tubes (correlates to the basolateral side of the Transwell™ insert). On day 22, the inserts were re-inverted: The silicone tubes were removed, and the inserts taken off the box and placed again into the original 12-well Transwell™ plate. On day 23, cells were exposed to 10 ng/mL LPS from the basolateral side to provoke an inflammatory response (or cultured without LPS as non-inflamed model). On day 24, it is designated to incubate the co-culture with nanoparticles as well as other substances, and cells can be harvested and analyzed with the desired methods on day 25.

### Co-culture application on particles

To verify the applicability of the co-culture models, the system was exposed to plastic particles of different sizes and materials. In detail, the models were incubated with either PLA2000, PLA250, MF366, or PMMA25 as described and characterized in the methods Section (2.2) for 24 h from the apical side on day 24 of the co-culture protocol. Concentrations of 2.5 × 10^9^ µm^2^ particle surface/mL were used. This amount has already shown to be not cytotoxic to Caco-2 cells [29]. We have chosen this unit for incubation because it ensures comparability between particles of different sizes, since the relative surface of particles increases with decreasing size.

### TEER measurements and FITC-dextran leakage

The integrity of the Caco-2 monolayer was checked in all experiments using two independent methods, transepithelial electrical resistance (TEER) measurements and fluorescein isothiocyanate-dextran (FITC-dextran) leakage. TEER was measured using an EVOM2 epithelial voltohmmeter (World Precision Instruments, Sarasota, USA). Paracellular permeability was assessed using 10 kDa FITC-dextran diluted in DMEM + . FITC-dextran was added to the apical chamber in two wells of each Transwell™ experiments at a concentration of 1 mg/mL. Afterwards, samples from the apical and basolateral chambers were collected and analyzed for FITC by fluorometry using the plate reader Infinite® M200Pro (Tecan Group Ltd., Männedorf, Switzerland) at an excitation of 485 nm and an emission of 535 nm. The apparent permeability coefficients (*P*_app_) were calculated as described in a previous publication [36]. Values > 600 Ω × cm^2^ and *P*_app_ < 10^7^ cm/s on day 23 of the co-culture confirmed the integrity of the Caco-2 monolayer.

### Real-time quantitative PCR (RT qPCR)

For evaluating the differentiation and inflammatory markers expressed during the simulation experiments, RT qPCR was performed. Therefore, the cell suspensions were collected and centrifuged at 130 × *g* for 8 min. The cell pellet was lysed with lysis buffer consisting of RLT buffer (part of the RNeasy Mini Kit) and 1% β-mercaptoethanol. For RNA isolation, the RNeasy Mini Kit (Qiagen GmbH, Hilden, Germany) was applied according to the manufacturer’s instructions. The final amount of extracted RNA was quantified with a NanoQuant® plate in the plate reader at Ex/Em 260/280 nm. For cDNA synthesis, the high-capacity cDNA reverse transcription kit (Applied Biosystems, Waltham, USA) was used as described by the manufacturer. Depending on the amount of RNA in the sample, the volume of nuclease-free water was adjusted to a final volume of 6.6 µL. In total, 1000 ng RNA per sample was used. The cDNA was amplified in a GeneAmp PCR system 9700 thermocycler (Applied Biosystems, Waltham, USA). Afterwards, the samples were diluted to a final cDNA concentration of 20 ng/µL in nuclease-free water. The gene expression of the genes depicted in Table 1 was measured on a 7900HT fast real-time PCR system (Applied Biosystems, Waltham, USA) using the Maxima SYBR Green/ROX qPCR Master Mix (Thermo Fisher Scientific, Waltham, USA). The genes *HPRT1* and *GAPDH* were used as housekeeping genes and values were averaged (Table 2). For analysis, the ∆∆C_t_ method was applied [37]. Undifferentiated cells and/or untreated cells served as controls.

**Table 2:** List of applied genes and their respective forward and reverse sequences

Gene	Sequence
*GAPDH*	Forward: GTCAAGGCTGAGAACGGGAA Reverse: AAATGAGCCCCAGCCTTCTC
*HPRT1*	Forward: ACGTCTTGCTCGAGATGTGA Reverse: GAGCACACAGAGGGCTACAA
*CD14*	Forward: AAGCACTTCCAGAGCCTGTC Reverse: TCGTCCAGCTCACAAGGTTC
*CD1a*	Forward: GCCACATCAGACTTGTTCCA Reverse: CTCTCCTTGTCACCAACCTCC
*CD209*	Forward: CTCCATCACCGCCTGCAAAG Reverse: GCTCCTCAGCACTTTTGATTACG
*CD80*	Forward: TTCTGTTCAGGTGTTATCCACG Reverse: TGCCAGTAGATGCGAGTTTGT
*CD86*	Forward: TCGCAACTCTTATAAATGTGGAACC Reverse: CACGCTGGGCTTCATCAGA
*IL-10*	Forward: CGAGATGCCTTCAGCAGAGTG Reverse: CTTGGCAACCCAGGTAACCCTTA
*IL-8*	Forward: GACATACTCCAAACCTTTCCACC Reverse: AATTTCTGTGTTGGCGCAGTG
*TNFA*	Forward: CTGGGCAGGTCTACTTTGGG Reverse: GAGCCAGAAGAGGTTGAGGG
*IL-6*	Forward: GAGTCTACCCAGCCCTTGTT Reverse: ACCATCACCGCCATCTACAT

### Staining and confocal microscopy

For some experiments, MUTZ-3 and THP-1 cells were stained before they were seeded on the basolateral side of the Transwell™ membrane on day 21. As dye, the CellTrace™ Cell Proliferation Kits (Invitrogen, Waltham, USA) violet and far red were used according to the manufacturer’s instructions. On day 25, the Transwell™ membranes were fixed with 3.7% formaldehyde diluted in PBS at 37 °C. Afterwards, membranes were washed three times with PBS and cells permeabilized with 0.2% Triton-X 100 diluted in PBS for 20 min at room temperature under light exclusion. Membranes were washed again three times with PBS. To further visualize the intestinal barrier, the co-culture membranes were stained with 2 drops/mL ActinGreen™ 488 ReadyProbes™ Reagent (Life Technologies, Carlsbad, USA) in PBS for 30 min at room temperature under light exclusion according to the manufacturer’s instructions. Again, membranes were washed three times with PBS. For embedding, the membranes were excised off the insert with a scalpel and placed on microscope slides. The samples were mounted using Kaiser’s glycerin gelatin and lid with cover glasses. After drying the slides overnight at 4 °C, the interaction of the immune cells with the intestinal barrier was investigated by using the laser scanning confocal microscope LSM 700 (Carl Zeiss AG, Oberkochen, Germany). The prepared samples were analyzed using the XYZ acquisition mode, × 40 objective, and contrast adjustment. Using this mode, the sample is depicted from its lateral side. Z-stacks were recorded starting from the villi of the Caco-2 cells (apical side) and ending beneath the membrane, where the immune cells were seeded (basolateral side). For each independent experiment (*n* = 3), 4 to 5 randomly selected sections of the membrane were analyzed.

### Cell counting in the basolateral compartment

To assess movement of the immune cells, the numbers of MUTZ-3-derived dendritic cells and THP-1-derived macrophages present in the basolateral compartment after inflammation with LPS were quantified, for co-culture as well as for triple-co-culture models. Therefore, the cell cultures were incubated on day 23 with or without LPS as described before, and the basolateral cell culture medium was harvested on day 24. The samples were centrifuged at 120 × *g* for 7 min and the cell pellets resuspended in 500 µL PBS. After mixing 10 µL of the resuspended sample with 10 µL 0.4% Trypan Blue solution (Lonza Group AG, Basel, Switzerland), 10 µL was pipetted in a counting slide and the cell concentration determined by an LUNA-II™ Automated Cell Counter (Logos Biosystems, Gyeonggi-do, South Korea).

### Cytokine release

For the quantification of cytokine release of the immune cells after inflammation or particle incubation, a Luminex® FLEXMAP 3D (Luminex Corporation, Austin, Texas) was used. The immune cell lines THP-1 and MUTZ-3 were differentiated as described in the methods Section (2.4). Next, cells were incubated for 24 h with 10 ng/mL LPS and subsequently incubated with respective nano-, submicro-, or nanoplastic particles for 24 h. The supernatant was harvested and directly used for further steps. For sample preparation, the Invitrogen™ (Invitrogen, Waltham, USA) ProcartaPlex™ Human Basic Kit (cat.no.: EPX010-10,420–901), the IL-6 Human ProcartaPlex™ Simplex Kit (cat.no.: EPX01A-10213–901), the IL-8 (CXCL8) Human ProcartaPlex™ Simplex Kit (cat.no.: EPX01A-10204–901), the TNF alpha Human ProcartaPlex™ Simplex Kit (cat.no.: EPX01A-10223–901), and the IL-10 Human ProcartaPlex™ Simplex Kit (cat.no.: EPX01A-10215–901) were used according to the manufacturer’s instructions. The data were analyzed using the ProcartaPlex™ analysis app. In short, cytokine amounts were quantified in picograms per milliliter based on a standard curve for each cytokine with a 5PL logarithmic curve fitting.

### Statistical analysis

Statistical analyses were performed using SigmaPlot 14.0 (Systat Software GmbH, Erkrath, Germany) or R version 4.1.0 (R Core Team, 2020). For data related to establishing a suitable MUTZ-3 differentiation protocol, one-way ANOVA followed by the Holm-Sidak test was applied. For data from the simulation experiments and cytokine release, the one-way Welch test followed by Games-Howell post hoc test was used, if the Kruskal–Wallis test for equal variance and the Shapiro–Wilk test for normality failed. Results of the treatment groups (differentiated cells) were compared to control groups (proliferating cells) and/or including LPS stimulation.

## Results

### Co-culture establishment

**Fig. 5:**
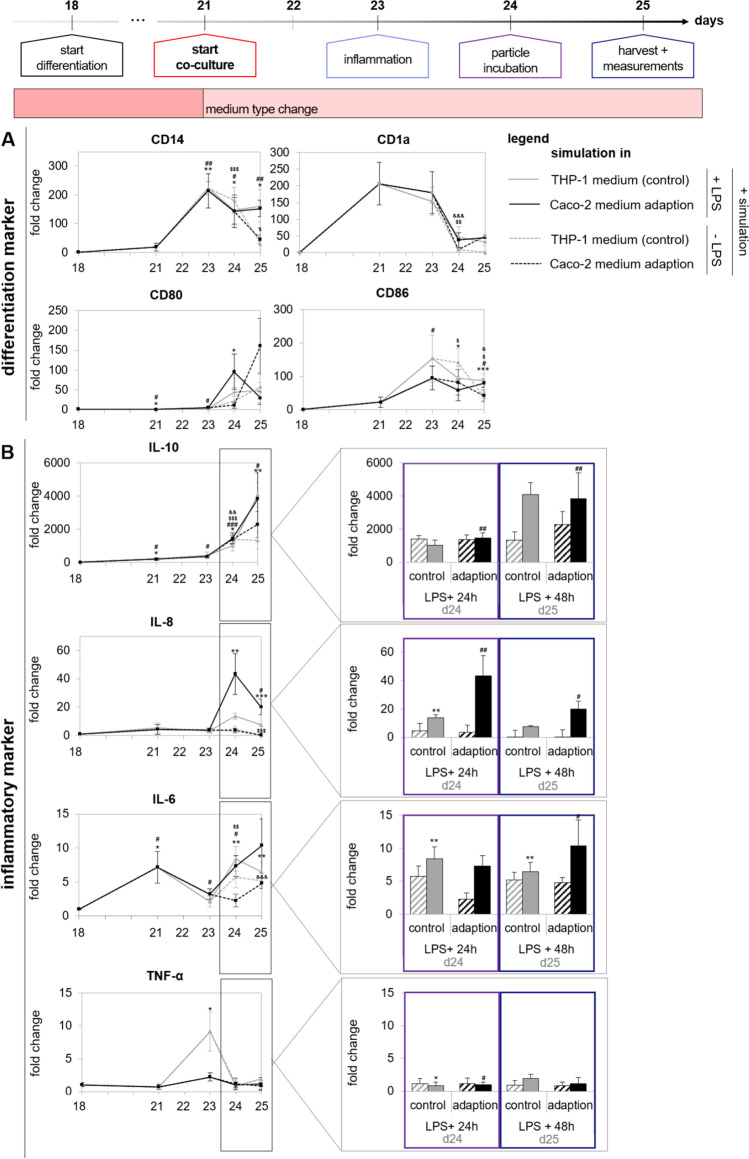
Expression and maintenance of THP-1-derived M0-macrophage differentiation and inflammatory markers, as quantified by RT qPCR during the time course of co-cultivation under different medium conditions (control = RPMI + or adaption = medium change to DMEM + after differentiation). **A** Quantification of differentiation markers CD14, CD1a, CD80, and CD86 in both medium conditions and with/without LPS incubation. **B** Quantification of inflammatory markers IL-10, IL-8, IL-6, and TNF-α in both medium conditions and with/without LPS incubation. Data are presented as means ± SD, *n* = 3. Significance was calculated by one-way Welch test followed by Games-Howell test in comparison to proliferating cells. The signs * and # equal simulation with/without LPS under control conditions, $ and & equal simulation with/without LPS under adapted conditions (*^,#,$,&^*p* ≤ 0.05; **^,##,$$,&&^*p* ≤ 0.001; ***.^,###,$$$,&&&^*p* < 0.001)

Figure 5, supplementary Figure S1, and supplementary Table S1 give the microscopic and transcription marker results for the introduction of THP-1-derived M0-macrophages to the co-culture surrounding, and over the time course of co-cultivation. After differentiation, the THP-1 cells exhibited morphological properties of immature M0-macrophages and became adherent. During the co-culture simulation, the morphology of the cells did not change depending on the time and culture medium. Additionally, the gene expression of the differentiation markers *CD14*, *CD1a*, *CD80*, and *CD86* was quantified on days 21, 23, 24, and 25. Only expression of *CD1a* was upregulated directly on day 21. The gene expression of the other markers significantly increased on day 23 and was maintained until day 25. For CD14, a significant upregulation was quantified on days 24 and 25 with LPS incubation in comparison to non-stimulated cells. There were no noticeable differences in gene regulation between the two medium conditions and with/without LPS stimulation for other markers. The pro- and anti-inflammatory properties of THP-1 cells associated with the polarization into M1-macrophages were assessed by investigating the genes *IL-10*, *IL-8*, *IL-6*, and *TNFA* and the results are depicted in Fig. 5B*.* All measured genes except *TNFA* were significantly upregulated on days 24 and 25 in comparison to the unstimulated cells. Especially, the anti-inflammatory cytokine *IL-10* was strongly expressed on day 24 and furthermore increased on day 25. The anti-inflammatory cytokine *IL-8* was especially upregulated on day 24, but again downregulated already on day 25. For *TNFA*, no significant response to the LPS stimulus was quantified. Again, no noticeable differences between the medium conditions were quantified. The results underline that THP-1-derived M0- and M1-macrophages withstand the co-cultivation duration and the medium type change to DMEM + , and are able to react to a pro-inflammatory stimulus.

**Fig. 6:**
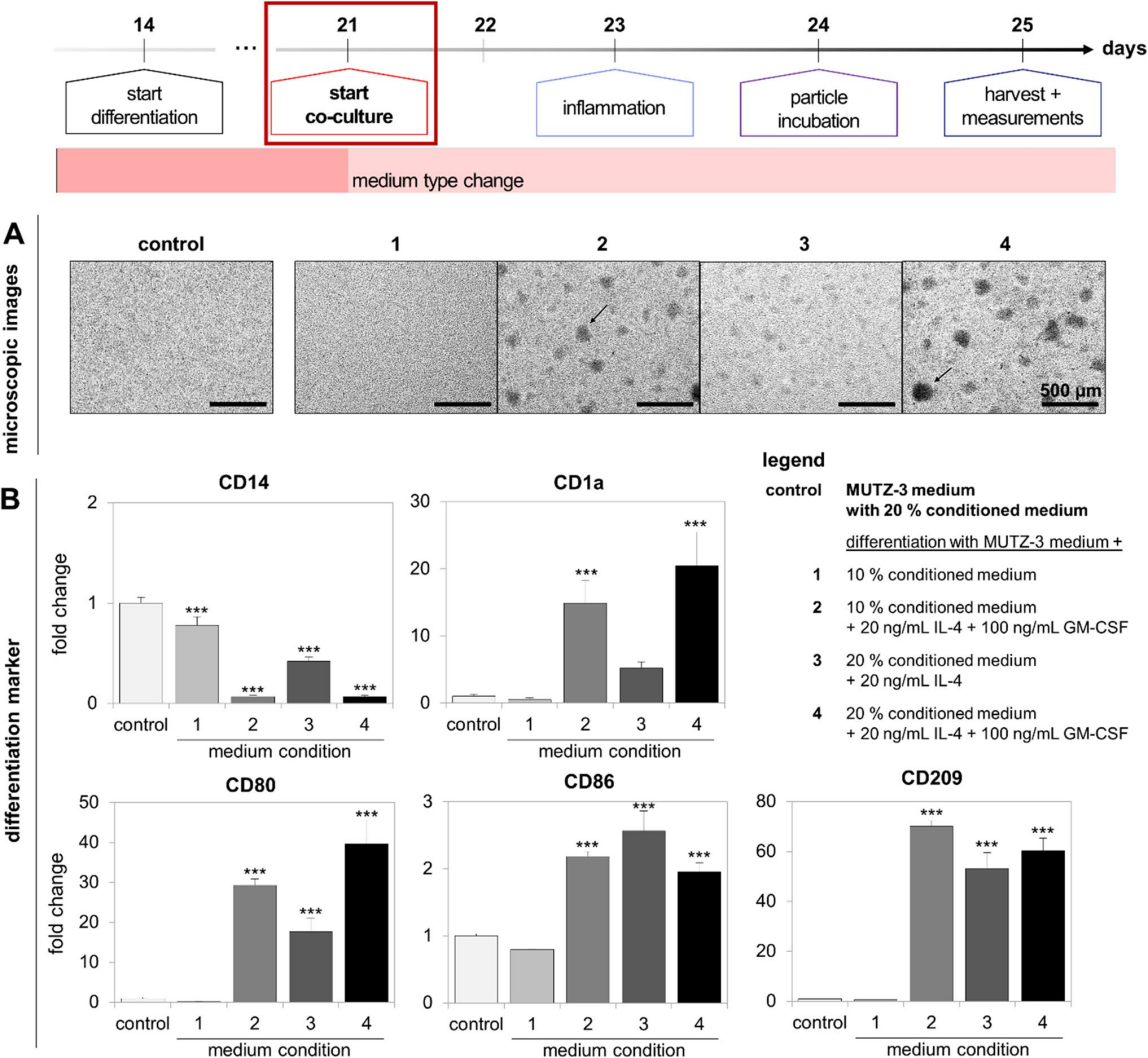
Development of a suitable MUTZ-3 differentiation protocol into immature dendritic cells by monitoring morphological changes and analyzing gene expression patterns. Four conditions were tested as given in the figure. Cytokines were refreshed on the third day of differentiation. **A** Microscopic examination of differentiation conditions on day 7. Images were taken at × 50 magnification. The scale bar displays a length of 500 µm. Desired cell clot formation is indicated by arrows. **B** Analysis of gene expression of the differentiation markers CD14, CD209, CD80, CD86, and CD1a by RT qPCR in harvested cell samples. Data are presented as means ± SD, *n* = 3. Significance was calculated by one-way ANOVA followed by Holm-Sidak test (***p < 0.001)

**Fig. 7:**
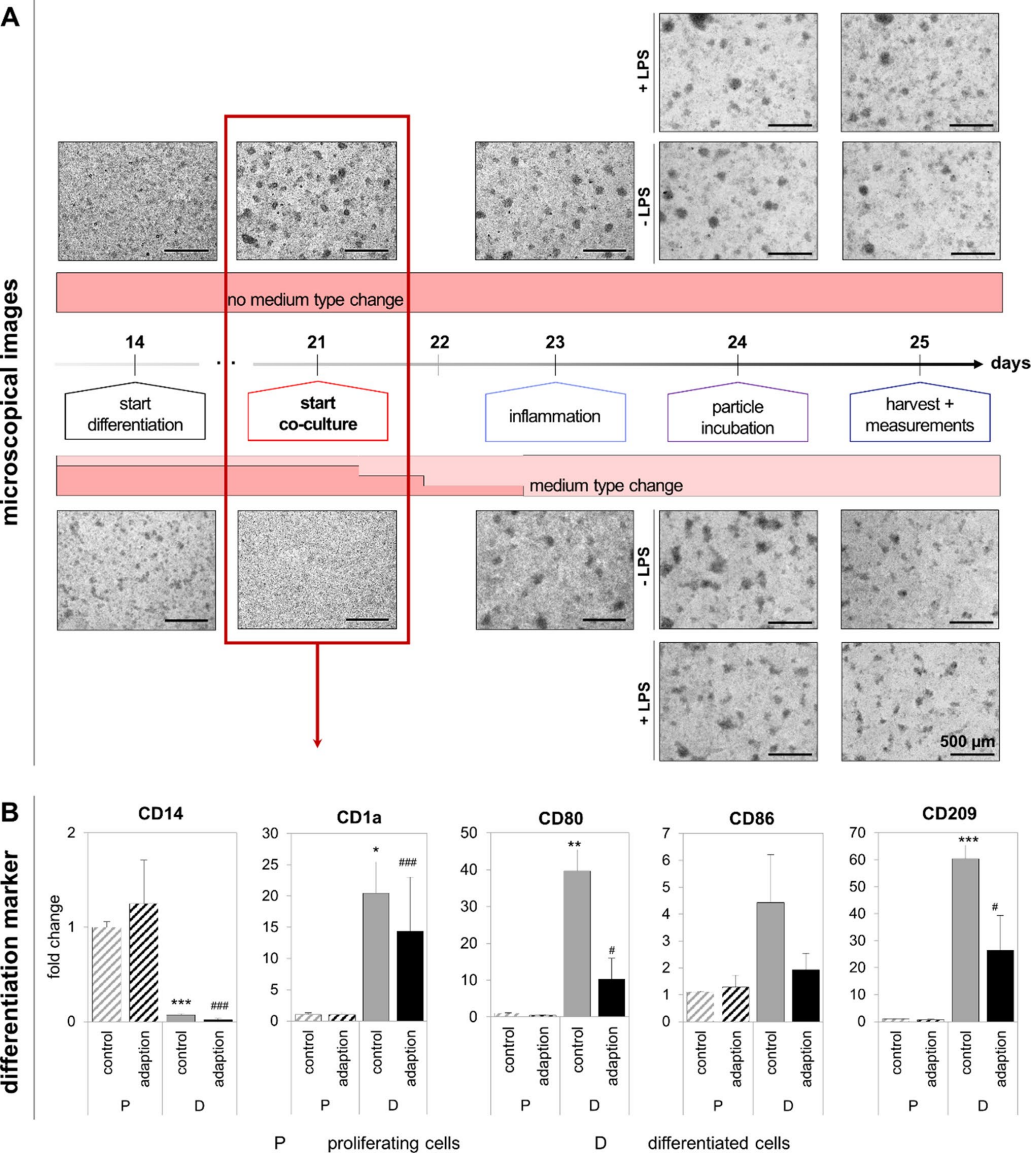
Monitoring morphological differences and differences in differentiation markers of MUTZ-3 cells under different medium conditions (control = MEM + or adaption = medium change to DMEM +). **A** Microscopic examination of MUTZ-3 cells during differentiation and co-culture procedure. Upper images show cells without medium type change and lower images with medium type change. Images were taken with a × 50 magnification. The scale bar displays a length of 500 µm. **B** Analysis of gene expression of the differentiation markers CD14, CD209, CD80, CD86, and CD1a by RT qPCR under both medium conditions. Data are presented as means ± SD, *n* = 3. Significance was calculated by one-way Welch test followed by Games-Howell test in comparison to proliferating cells (*^,#^*p* ≤ 0.05; **^,##^*p* ≤ 0.001; ***^,###^*p* < 0.001)

In parallel to the adaption of THP-1 cells, appropriate differentiation of MUTZ-3 cells to immature dendritic cells and their integration into the co-culture model were analyzed; the results are given in Figs. 6 and 7. First, the results for the selection of the most suitable differentiation protocol are depicted in Fig. 6. Based on the differentiation protocol by Nyambura et al., four different differentiation conditions were tested and compared to the control condition MEM + with 20% conditioned medium of 5637 cells: differentiation in MEM +  + (1) 10% conditioned medium, (2) 10% conditioned medium + 20 ng/mL IL-4 + 100 ng/mL GM-CSF, (3) 20% conditioned medium + 20 ng/mL IL-4, (4) 20% conditioned medium + 20 ng/mL IL-4 + 100 ng/mL GM-CSF [35]. After differentiation, cells were examined using microscopy (Fig. 6A) and analysis of gene expression of the dendritic cell markers *CD14*, *CD209*, *CD80*, *CD86*, and *CD1a* (Fig. 6B). Microscopic examination of MUTZ-3-derived dendritic cells showed that cells cultivated in conditioned medium only (control and condition 1) exhibited similar morphologies. Upon treatment of cells with IL-4 and/or GM-CSF (condition 2–4), a cell clot formation (indicated by arrows) was observed. Accompanying this observation, an increased cell adhesion was detected. The size and amount of adherent cells were most prominent in condition 4. The comparison of gene expression between the different conditions (Fig. 6B) confirmed the microscopic observations. Comparing the control and condition 1, almost no significant differences of gene regulation were measured. Only for *CD14*, a small but significant downregulation was observed. The conditions 2 to 4 exhibited significant differences in gene expression in comparison to the control. Upon treatment of cells with IL-4, *CD14* was significantly downregulated. The genes *CD209*, *CD80*, *CD86*, and *CD1a* were significantly upregulated. Especially conditions 2 and 4, where cells were additionally treated with GM-CSF, showed the strongest effects in comparison to the control. Regarding conditions 2 and 4, no significant differences between the genes were detected. Nevertheless, the medium composition in condition 4 is more closely related to the recommended medium conditions of MUTZ-3 cells, since it comprises 20% conditioned medium. Therefore, the most suitable differentiation protocol was regarded to be as follows: MEM + with 20% conditioned medium of 5637 cells + 20 ng/mL IL-4 + 100 ng/mL GM-CSF for a differentiation time of 7 days and a cytokine refresh on the third day. These conditions were used for all following experiments.

Results for the second step, the adaption of MUTZ-3 cells to the co-culture conditions, are given in Fig. 7. The expressions of the differentiation markers, as well as the inflammatory response and morphological changes, were monitored by RT qPCR and fluorescence microscopy. Importantly, initial experiments showed that the survival of differentiated MUTZ-3 cells that received a medium change to Caco-2 co-culture medium in one single step was very low during the period of co-culture time. More information can be found in the supporting Figure S2. In order to improve the survival of dendritic-like cells, cells were gradually adapted to DMEM + before and during differentiation. Figure 7 illustrates morphological changes of MUTZ-3 cells during differentiation and simulation of the co-culture workflow under control conditions (cultivation in MEM + during differentiation and simulation) and under adapted conditions (stepwise DMEM + adaption during differentiation and simulation in DMEM +). In comparison to the control conditions, adapted MUTZ-3 cells formed characteristic cell clots initially on day 22, but maintained the morphology during the co-cultivation procedure. The size of cell clots increased from day 24, but did not change between inflamed or non-inflamed conditions, or between different medium conditions. The expressions of differentiation markers after DMEM + adaption in comparison to proliferating cells and normally differentiated cells are shown in Fig. 7. The monocyte marker *CD14* was significantly downregulated and significant upregulation of the genes *CD1a*, *CD80*, *CD86*, and *CD209* was quantified under both differentiation conditions. Even though the medium adaption diminished the degree of marker gene expression differences compared to undifferentiated proliferating cells, gene expression was still significantly different compared to proliferating cells.

**Fig. 8:**
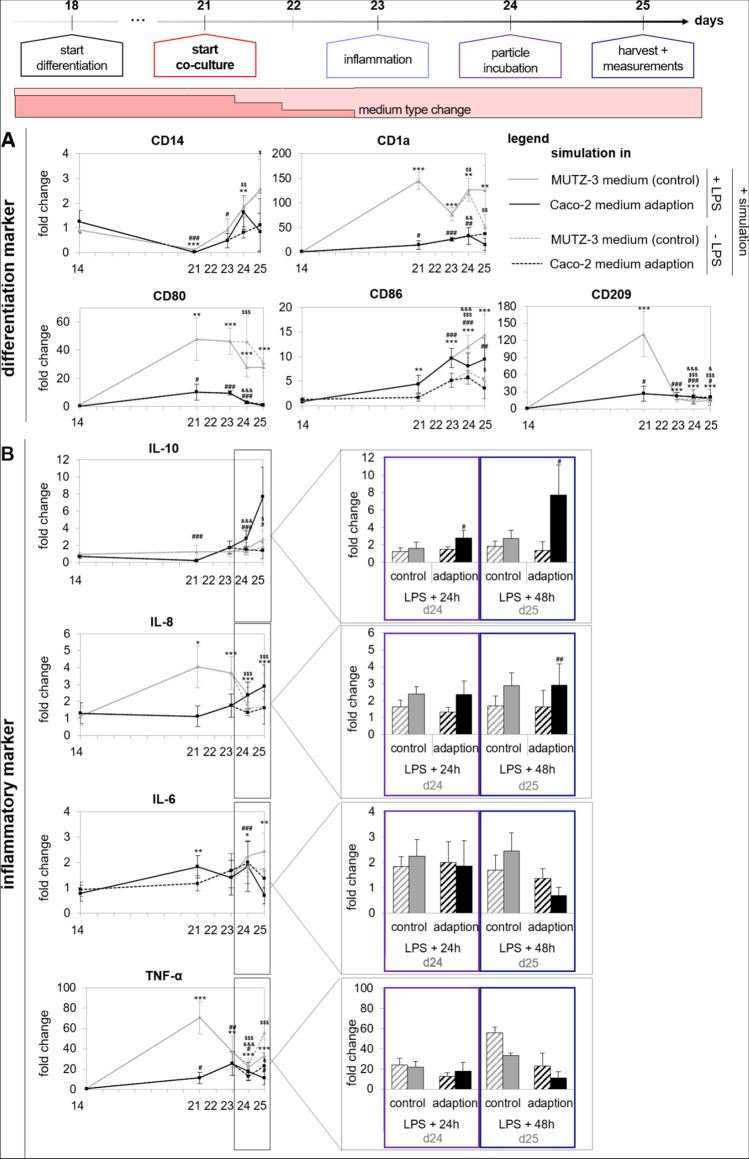
Expression and maintenance of MUTZ-3-derived dendritic cell differentiation and inflammatory markers, quantified by RT qPCR during the time course of co-cultivation under different medium conditions (control = MEM + , or adaption = medium change to DMEM +). **A** Quantification of the differentiation markers CD14, CD1a, CD80, CD86, and CD209 in both medium conditions and with/without LPS incubation. **B** Quantification of inflammatory markers IL-10, IL-8, IL-6, and TNF-α in both medium conditions and with/without LPS incubation. Data are presented as means ± SD, *n* = 3. Significance was calculated by one-way Welch test followed by Games-Howell test in comparison to proliferating cells. The signs * and # equal simulation with/without LPS under control conditions, $ and & equal simulation with/without LPS under adapted conditions (*^,#,$,&^*p* ≤ 0.05; **^,##,$$,&&^*p* ≤ 0.001; ***.^,###,$$$,&&&^*p* < 0.001)

In Fig. 8, the maintenance of differentiation markers, basal cytokine production, and inflammatory responses of MUTZ-3-derived dendritic cells during the simulation of the co-cultivation procedure are depicted. The exact values of the gene expression analyses can be found in supplementary Table S1. As already shown in Fig. 7, all investigated differentiation markers were significantly downregulated (*CD14*) or upregulated (*CD1a*, *CD80*, *CD86*, and *CD209*). The most prominent upregulation was quantified for *CD1a*, *CD80*, and *CD209* and was maintained until day 25. Between the two medium conditions, some slight differences were observed for *CD80* and *CD1a*. The gene expression under both medium conditions was significantly different from proliferating MUTZ-3 cells. The pro- and anti-inflammatory properties of MUTZ-3 cells after LPS stimulation were assessed by investigating the genes *IL-10*, *IL-8*, *IL-6*, and *TNF-α* in comparison to unstimulated cells and the results are depicted in Fig. 8B*.* The pro- and anti-inflammatory cytokines *IL-8* and *IL-10* were significantly upregulated especially under adapted medium conditions. For *IL-6*, an upregulation was only quantified for control conditions, but not in the adapted cells. TNF-α was not upregulated in any of the tested conditions. For the inflammatory markers, no noticeable differences in gene regulation on days 21 and 23 were observed. With our adapted differentiation protocol, we were able to demonstrate that MUTZ-3-derived dendritic cells are able to maintain their differentiation status during co-cultivation and are able to get activated after addition of a pro-inflammatory stimulus.

**Fig. 9:**
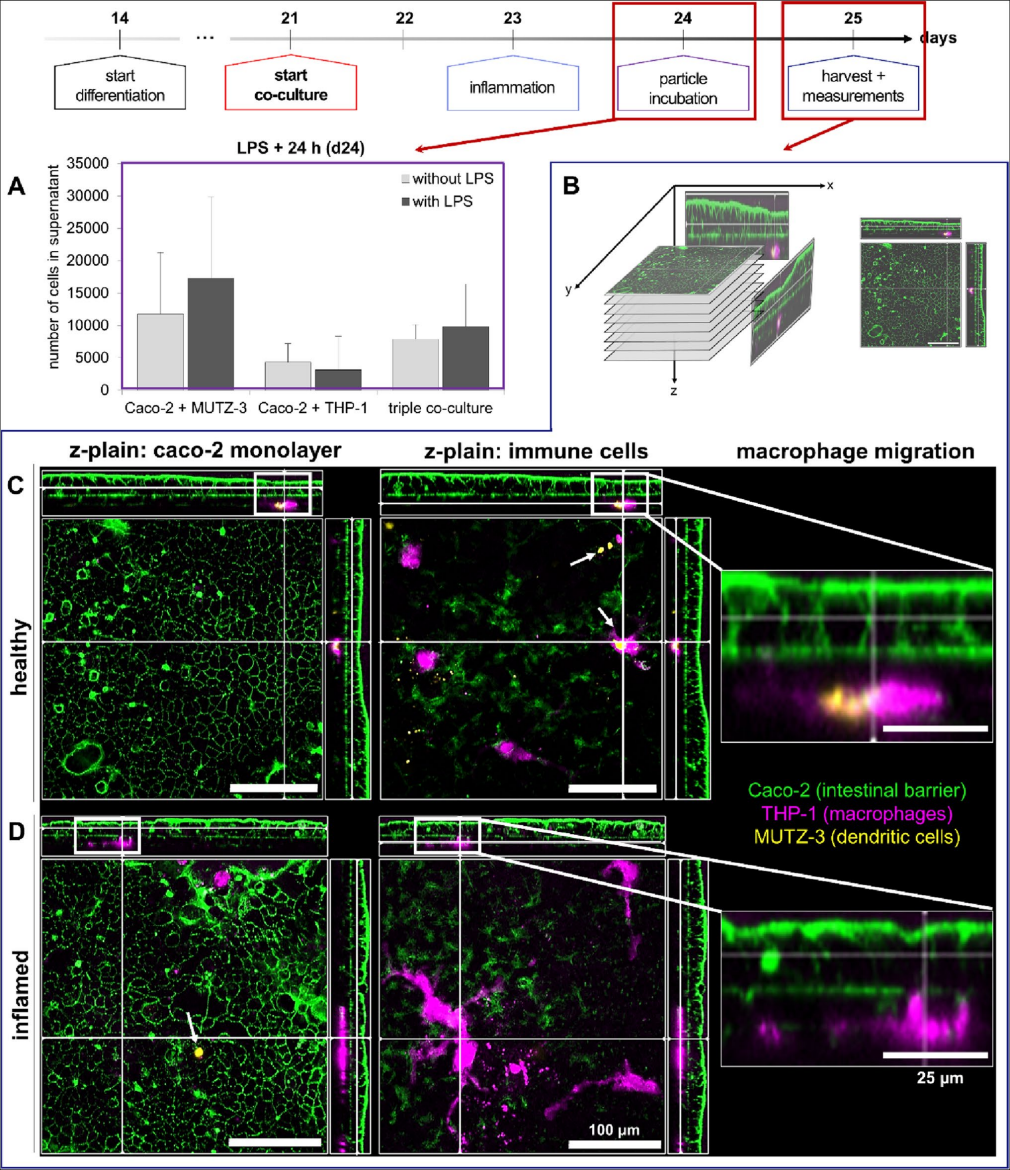
Migration of MUTZ-3-derived dendritic cells and THP-1-derived macrophages in the final co-culture model of the healthy and inflamed intestinal immune system. **A** Number of cells counted in the basolateral compartment on day 24, 24 h after inflammation with LPS. **B** Schematic depiction of z-stack imaging. **C**, **D** Representative confocal microscopy examinations of the final co-culture models of the healthy (**C**) and inflamed (**D**) intestinal immune system. The Caco-2 monolayer is depicted in green, the THP-1-derived macrophages are depicted in pink, and the MUTZ-3-derived dendritic cells are shown in yellow. Left: z-plain of the Caco-2 monolayer, middle: z-plain of the immune cells. Both scale bars display a length of 100 µm. Right: enlarged section of the macrophages to substantiate M1-macrophage migration. The scale bar displays a length of 25 µm

Finally, after the functionality of the immune cells had been proven in simulation experiments, the final co-culture setup was posed as depicted in Fig. 9 by combining all three cell lines in direct cell–cell contact. In this step, the functionality and characteristics of the Caco-2 monolayer and the immune cells in the healthy and inflamed model were monitored by counting cells in the basolateral compartment after potential LPS inflammation (Fig. 9A), via confocal microscopy (Fig. 9B–D), TEER barrier integrity analysis (Figure S3A), and FITC-dextran leakage (Figure S3B). The integrity measurements did not show differences in the tightness of the Caco-2 monolayer between the healthy and inflamed models. This observation was substantiated by confocal microscopy (Fig. 9C, D). The images further confirmed the presence of macrophages (THP-1, pink) and dendritic cells (MUTZ-3, yellow) in close contact to the intestinal monolayer (Caco-2, green) from its basolateral side. After inflammation with LPS, the M1-macrophages changed their morphology and size in comparison to the healthy state. The cells exhibited an irregular shape and started migrating into the intestinal barrier as a reaction to the proinflammatory stimulus. In addition to the confocal images, the amount of cells present in the basolateral compartment on day 24 (24 h after LPS inflammation) was counted in three different approaches: (1) co-culture model of differentiated Caco-2 cells and MUTZ-3-derived dendritic cells, (2) co-culture model of differentiated Caco-2 cells and THP-1-derived macrophages, and (3) triple co-culture model. In approaches (1) and (3), the amount of cells in the basolateral compartment increased after inflammation with LPS in comparison to non-inflamed samples. In contrast, approach (2) showed that the amount of cells decreased after inflammation. Therefore, the designated co-culture model was finally established and ready to use for particle research.

### Co-culture application on particles

**Fig. 10:**
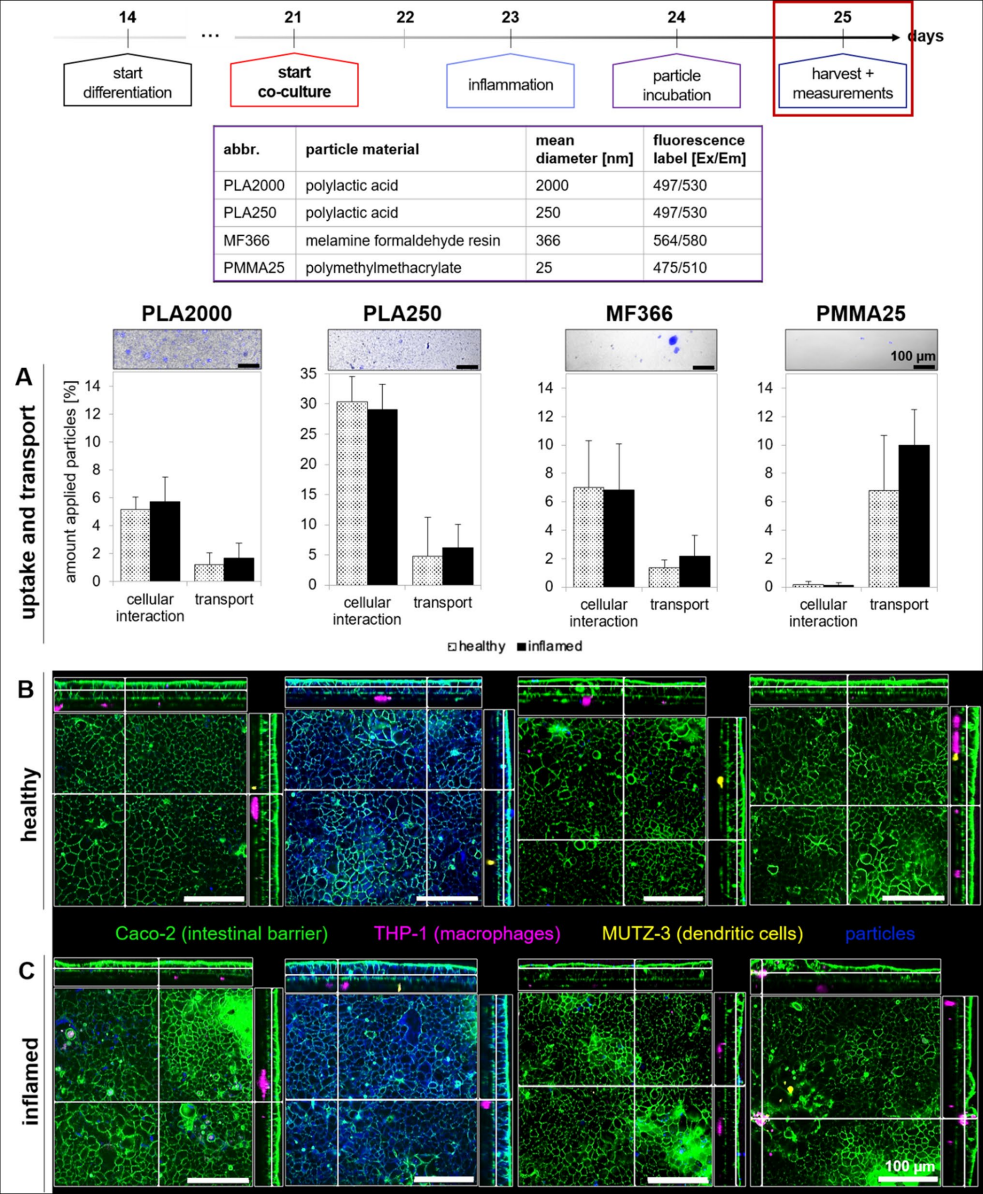
Cellular interaction and transport of PLA2000, PLA250, MF366, and PMMA25 after 24-h incubation with the healthy or inflamed co-culture model. **A** Quantification of cellular interaction and transport of particles in the healthy and inflamed model via fluorescence measurements of the particles present in the apical and basolateral compartment as well as on the Transwell™ membranes. Data are presented as means ± SD, *n* = 3. **B**, **C** Representative confocal microscopic examinations of particle interactions in the healthy and inflamed co-culture models. The Caco-2 monolayer is depicted in green, the THP-1-derived macrophages are depicted in pink, the MUTZ-3-derived dendritic cells are shown in yellow, and the particles are depicted in blue. The scale bar displays a length of 100 µm

Since the overall goal of the co-culture development was to use it for studies on nano- and microparticles, the model was applied to such particles made of different plastic materials: PLA2000, PLA250, MF366, and PMMA25. The cellular interaction and transport of these particles were measured via fluorescence quantification (Fig. 10A) in comparison to the healthy and inflamed medium control, and with confocal microscopy (Fig. 10B). For all particles, except for PMMA25, a cellular interaction could be quantified, which was especially distinct for the submicrometer particles. Between the healthy and inflamed states, no significant differences were detected. The transport of particles slightly increased in the inflamed model compared to the healthy model. Especially for PMMA25, the transport increased from 6 to 9% in the inflamed setup. In the confocal images, the particles are depicted in blue. In particular, PLA250 was present inside the Caco-2 cells, allocated all over the inner surface. Particle interaction of PLA2000 and MF366 can also be seen. No interaction of plastic particles with the immune cells was observed. To prove whether nano-, submicro-, or microplastics activated immune cells, the release of cytokines was quantified.

**Fig. 11:**
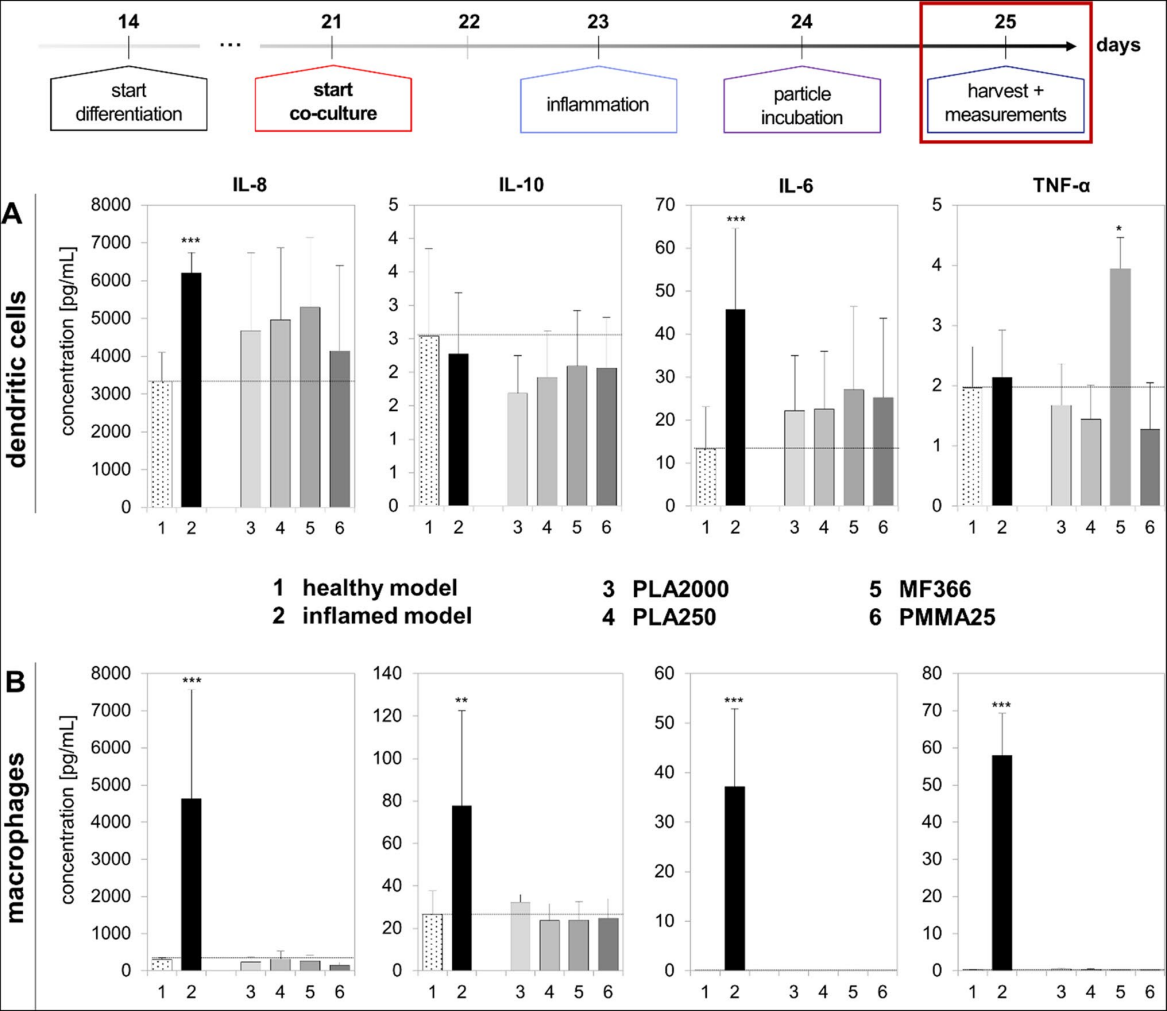
Cytokine release measurements of IL-8, IL-10, IL-6, and TNF-α quantified with a multiplex assay in MUTZ-3-derived dendritic cells (**A**) and THP-1-derived macrophages (**B**). The changes of pro- and anti-inflammatory cytokines were quantified for the healthy (1) and inflamed (2) models as well as for PLA2000 (3), PLA250 (4), MF366 (5), and PMMA25 (6). Data are presented as means ± SD, *n* = 3. Significance was calculated by one-way Welch test followed by Games-Howell test in comparison to medium control (*p ≤ 0.05; **p ≤ 0.001; ***p < 0.001)

The release of inflammatory markers of the immune cells was already quantified using RT qPCR. To confirm the expression patterns at the protein level, the cytokine release of each, THP-1 and MUTZ-3 cells in monoculture, was quantified with a multiplex assay. Additionally, it was important to prove whether the immune cells get activated after incubation with plastic particles. The results are depicted in Fig. 11. For both cell lines, activation of the immune cells by LPS was proven by significant upregulation of the cytokines IL-8 and IL-6 in MUTZ-3 cells and IL-8, IL-10, IL-6, and TNF-α in THP-1 cells, as compared to the healthy model. The basal cytokine expression did not increase after particle incubation in THP-1 cells. MUTZ-3 cells upregulated the release of the pro-inflammatory cytokines IL-8 and IL-6 after 24 h exposure to all particle species. Only MF366 was able to increase the release of TNF-α in MUTZ-3 cells.

## Discussion

The intestinal immune system is the largest and most complex part of the immune system, forming a border between the body and the environment. Its homeostasis and fine balance are vital to its function since it has important functions in pathogen defense. Any perturbation can cause severe negative effects such as (chronic) inflammation, disrupted epithelial barrier function, and tissue damage. The development of an immune competent in vitro model of the small intestine is of great interest due to the rising incidence of intestinal disorders and the variety of ingested substances that need to be analyzed for their toxicological potential.

This study presents a new model for the healthy and inflamed human intestine. It combines the advantages and features of the well-established Caco-2 model for the intestinal epithelial enterocytes, THP-1-derived M0-macrophages, and newly adapted MUTZ-3-derived immature dendritic cells. The use of a Transwell™-based co-culture gives access to both sides of the epithelial barrier. It additionally provides the opportunity to investigate both particle transport as well as the influence of immune cells, to the particle fate, and the consequence of particle exposure to intraepithelial immune cells and their homeostasis.

During the last years, different co-culture models of the human intraepithelial immune system have been developed for different purposes. Diversity and progress of these attempts are described in review article by Ponce de León-Rodríguez [38]. Latest studies also present first steps to use such in vitro models for reducing animal experiments [39]. All models are based on the well-established intestinal cell line Caco-2, which was often co-cultivated with THP-1 cells. Especially for THP-1, available protocols are diverse regarding the inducing agent and its concentration, incubation time, and intended state of differentiation of macrophages (M0, M1, or M2) [40]. Attention should be paid to the fact that for our in vitro model the differentiation into an M0 phenotype is needed to investigate maturation upon stimulation with pro-inflammatory stimuli or to identify the influence of particles on this process [41–44]. We could demonstrate immune competence of THP-1-derived M1-macrophages by measuring an upregulation of four representative immune modulators (IL-10, IL-8, IL-6, and TNF-α) upon LPS stimulation. The pro-inflammatory marker IL-8 was strongly upregulated 24 h after LPS incubation and immediately downregulated 48 h after inflammation. This is consistent with the anti-inflammatory marker IL-10, which was initially upregulated 48 h after inflammation. It is proven that pro-inflammatory cytokines like IL-8 are downregulated by IL-10 to suppress pro-inflammatory immune response and to induce anti-inflammatory signaling [45, 46]. Kämpfer et al. (2017) also quantified an increased release of pro-inflammatory interleukins in IFN-γ/LPS-activated M1-macrophages [28]. Another advantage of THP-1 cells is their ability to express and maintain *CD14* during the whole co-culture time and respond to LPS stimuli with an upregulation of *CD14* as demonstrated by our experiments. Even though it does not display the situation in in vivo macrophages, which lack *CD14* expression [47], it makes our model more vulnerable to reveal potential induction of inflammation. This has to be kept in mind when interpreting the results.

In contrast, cell line-based models for dendritic cells are more rarely used. The standard source of dendritic cells are mainly monocyte-derived dendritic cells from different donors [48–50]. Nonetheless, due to the limited availability of such cells and their heterogeneity, our goal was to develop a protocol for the differentiation of dendritic cells derived from an immortal cell line that can be used in DMEM + medium. Comparing potential precursor cells applied for dendritic cell differentiation, namely THP-1, KG-1, and MUTZ-3 [48, 51], only the MUTZ-3 cell line fulfils all demands. MUTZ-3 cells are able to represent different maturation states that depend on the applied stimulus and to represent many morphologic, phenotypic, and functional properties of dendritic cells [35, 52–54]. Despite all the advantages of the MUTZ-3 cells, there are also a few drawbacks that should be mentioned here: Their handling is sophisticated since the MEM-based medium needs to be supplemented with 20% FCS and 20% supernatant of the 5637 cell line to ensure a sufficient presence of cytokines. This composition differs a lot from other well-established culture media such as for Caco-2 cells (DMEM +). A rapid medium change from MEM + to DMEM + resulted in prompt cell death as demonstrated in our studies. With our differentiation protocol that includes a medium adaption of MUTZ-3 cells to DMEM + , we were initially able to differentiate MUTZ-3 cells into an immature dendritic cell phenotype, to ensure survivability of these cells during co-cultivation with other cell lines and keeping the same incubation medium for the investigation on particles. We have shown the successful differentiation via an upregulation of the differentiation markers CD209, CD80, CD1a, and CD86. Additionally, the monocytic marker CD14 was downregulated, and CD86 was upregulated after LPS stimulation as a sign for maturation of dendritic cells. This is in concordance with other findings in the literature [48, 53, 54]. With cytokine release and RT qPCR measurements, we could show pro- and anti-inflammatory response of MUTZ-3-derived dendritic cells via upregulation of representative markers like IL-8 and IL-10 after LPS incubation. Susewind et al. also used MUTZ-3 cells in a co-culture model, but did not differentiate the cells or monitor expression of dendritic cell-like markers [27].

To fulfill our demands, the developed model combines differentiated Caco-2 cells on a Transwell™ membrane with PMA-derived THP-1 M0-macrophages and medium-adapted MUTZ-3 by inverting the Transwell™ insert for a short time period during the end of Caco-2 differentiation. This setup was implemented without adding a further biopolymer layer and therefore enables close cell–cell contact of immune and epithelial cells from the basolateral side of the epithelium. Earlier studies embedded immune cells in a collagen layer to prevent cell loss during co-cultivation [24, 27]. This approach yields disadvantages, such as limited transport across the intestinal barrier and restricted cell–cell contacts. Several other publications combined differentiated Caco-2 cells and PMA-differentiated THP-1 cells to evaluate either uptake of particles [26] or the cross-talk between intestinal cells and macrophages [25, 55, 56]. For this purpose, Caco-2 cells were differentiated on Transwell™ membranes and THP-1-derived macrophages were seeded on the well bottom beneath the Transwell™ insert. This approach even more restricts direct cell–cell contact of immune and epithelial cells. Only Mori et al. ensured direct cell–cell contact by developing an upside-down culture [56]. Caco-2 cells were seeded on the bottom side of a Millicell membrane and THP-1 cells were added to the inner chamber of the insert. In contrast to the other attempts and in line with our approach, this model is suitable to study migration of immune cells into the intestinal epithelium. However, it is not appropriate for studies with sedimenting substances like particles because they are not able to reach the intestinal cells. In our study, we switched the setting and the successful co-cultivation was proven by confocal microscopy. As a response to LPS stimulation, we showed the migration of THP-1-derived macrophages into the cell monolayer via confocal microscopy, while not detecting membrane integrity disruption. This is in line with the function of in vivo macrophages that are able to extend dendrites between intestinal epithelial cells to sample antigens [1]. Activated dendritic cells in vivo are suggested to migrate into the mesenteric lymph nodes to stimulate the production of immunoglobulin A and provoke a T cell response [57, 58]. By comparing the numbers of MUTZ-3-derived dendritic cells present in the basolateral compartment, we gathered evidence for an increasing migration of cells after stimulation with LPS.

The model presented here shall be used not only for experiments with soluble substances, but also with particle dispersions like nano- and microplastics. Such polymers require special attention to the incubation medium because they are characterized not only by their concentration like soluble chemicals, but also by their particle size distributions. Depending on the interaction of particles and the dispersion, specific characteristics like hydrodynamic diameter, protein corona, and dispersion stability can be different for the same particles [59]. Therefore, we adapted all cells to be cultivated in DMEM + , which allows direct comparability with other models. Then, we applied the model to nano- and submicroplastics from different polymers (PLA, MF, PMMA). We quantified transport and cytokine release triggered by these particles. In our previous study, where we used a Caco-2 mono-culture model, we measured a material- and size-dependent particle uptake [29]. Considering our newly developed model of the intestinal immune system, again a material- and size-dependent particle uptake and transport was observed, which was slightly higher than in the Caco-2 mono-culture. This difference can therefore be regarded to be caused by the influence of the immune cells and not by differences in the incubation medium. The results indicate that upon inflammation of the co-culture model with LPS, the transport of nano- and submicroplastic particles increases. Especially for PMMA25 as a representative for nanoplastics, the transport increased from 6.5 to 10% when comparing the healthy and inflamed states. Furthermore, the interaction of particles with the cells is material and size dependent, since the most pronounced interaction was quantified for the submicrometer particles and most notably for PLA250. Prior to our study, the following research groups used different co-culture models for investigation of plastic particle transport. Leonard et al. analyzed 50-nm polystyrene nanoparticles in differentiated Caco-2 and in co-culture with primary macrophages and dendritic cells in healthy and inflamed states [24]. As their goal was not a systematic analysis of the particle effect, they did not focus on the composition and comparability of the incubation medium used for the particles. They showed enhanced uptake of particles in immune competent cells in the inflamed model in comparison to the healthy model. In the same year, Moyes et al. incubated 2-µm latex particles on a co-culture of differentiated Caco-2 cells with PMA-derived M0-macrophages (THP-1) for 5 or 60 min [26]. This study was based on earlier findings, which showed that the tightness of Caco-2 tight junctions decreases after macrophage exposure [25]. Here, they did use only one culture medium for the co-culture as we did. A time-dependent increase of particle uptake transport across the intestinal barrier was observed compared to the Caco-2 monoculture and linked to the opening of the Caco-2 tight junctions upon THP-1 appearance. In our approach, the transport of plastic particles in the healthy co-culture did not increase in comparison to the Caco-2 monoculture. Since then, some studies have reported the development of inflammation and release of pro-inflammatory cytokines upon stimulation with nano- and microplastic particles, mainly polystyrene in vitro [60]. Prietl et al. exposed leukocytes derived from human blood, monocytes, and macrophages to carboxylated polystyrene nano- and submicroparticles (20 to 1000 nm diameter) [61]. They reported release of IL-8 in both monocytes and macrophages for all particles. Another group investigated the response of human gastric adenocarcinoma cells to 44-nm polystyrene particles and found upregulation of IL-6 and IL-8 [62]. Taken together, these results indicate potential effects of nano- and microplastic particles that might trigger the immune system. Nevertheless, these studies mainly used polystyrene, which is not the most abundant material type. For our test particles, we have shown a slight upregulation of IL-8 and IL-6 for all particles only in MUTZ-3-derived dendritic cells, but not in THP-1-derived M1-macrophages. Nevertheless, mechanisms of the emergence of inflammation remain unclear.

In conclusion, a small number of different co-culture models of the intraepithelial immune system of the intestine have been established previously, but they differ in complexity, composition, and function of their components, and they have been designed for different purposes. Especially research on small particles contains special needs in the handling and characterization of particle dispersions and design of the co-culture model to enable comparable and meaningful results, which are not always met by previous models. The co-culture model developed here enables investigation of particle transport and toxicity in the healthy and inflamed states.

## Supplementary Information

Below is the link to the electronic supplementary material.

Supplementary file1 (PDF 2564 KB)

## Data Availability

All data generated or analyzed during this study are included in this published article and its supplementary information files.

## Abbreviations

*DMEM*: Dulbecco’s modified Eagle medium
*EDTA*: Ethylenediaminetetraacetic acid
*FCS*: Fetal calf serum
*FITC*: Fluorescein isothiocyanate-dextran
*P/S*: Penicillin/streptomycin
*PBS*: Phosphate-buffered saline
*TEER*: Transepithelial electrical resistance
*RPMI*: Roswell Park Memorial Institute 1640
*α-MEM Eagle*: Alpha-modified minimum essential medium eagle
*RT qPCR*: Real-time quantitative polymerase chain reaction
*LPS*: Lipopolysaccharide
*IL-4*: Interleukin 4
*GM-CSF*: Granulocyte–macrophage colony-stimulating factor
*PLA*: Polylactic acid
*MF*: Melamine formaldehyde resin
*PMMA*: Polymethylmethacrylate
