# Supplementary material for: A human Caco-2-based co-culture model of the inflamed intestinal mucosa for particle toxicity studies
Source: In Vitro Model. 2023 Mar 24;2(1-2):43–64. doi: 10.1007/s44164-023-00047-y (PMC11756451; doi:10.1007/s44164-023-00047-y)
Supplement: Supplementary file 1 — Supplementary file1 (PDF 2564 KB) [file 44164_2023_47_MOESM1_ESM.pdf]

# SUPPORTING INFORMATION

# Caco-2 based co-culture model of the inflamed intestinal mucosa suitable for studies on particles

Maxi B. Paul, Marén Schlief, Hannes Daher, Albert Braeuning, Holger Sieg, Linda Böhmert*

German Federal Institute for Risk Assessment, Department of Food Safety, Max-Dohrn-Str. 8-10, 10589 Berlin, Germany

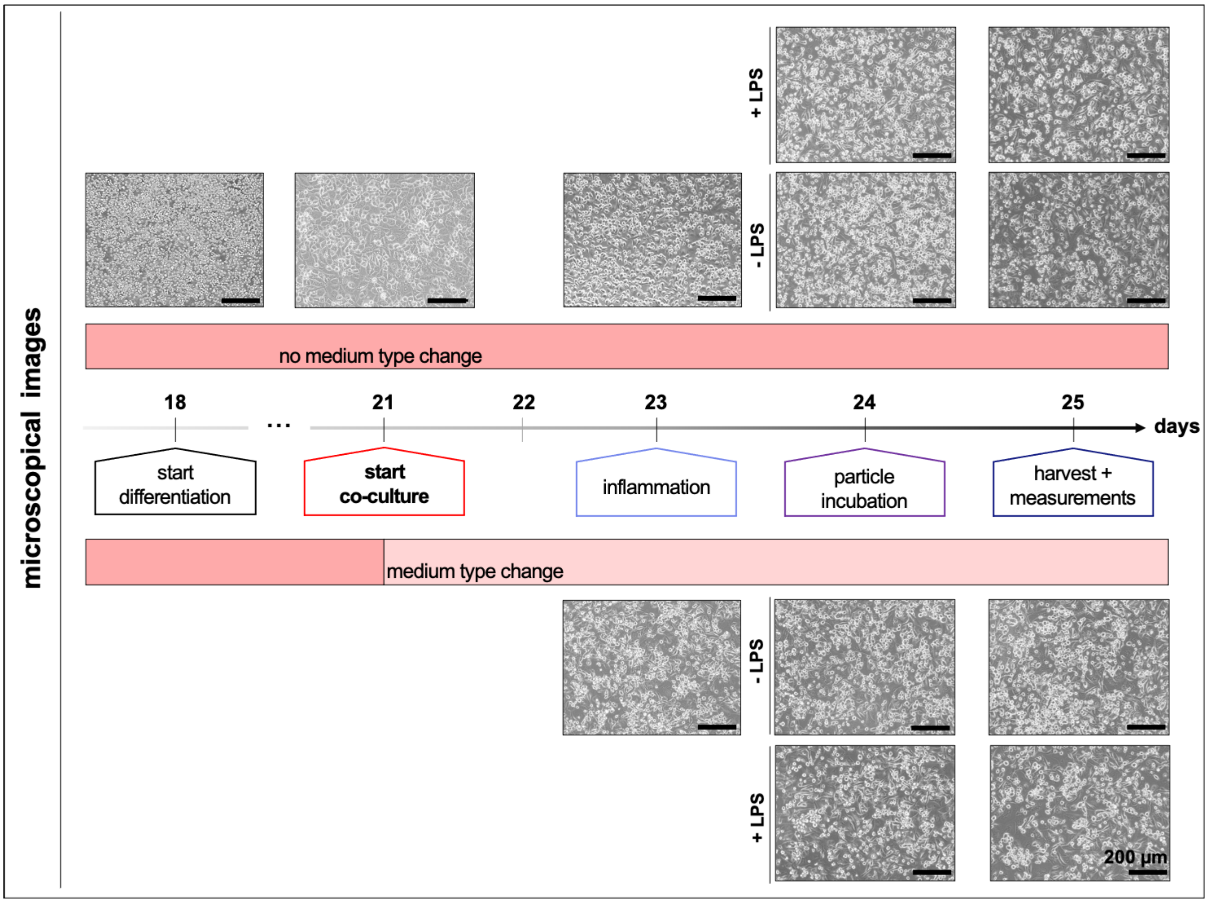


*Figure S 1: Microscopical examination of the morphologic changes of PMA-derived THP-1 M0-macrophages during the co-culture simulation procedure. The upper images represent cells without medium type change (only RPMI+). The lower images represent cells cultivated with medium type change from RPMI+ to DMEM+ on day 21. Images were taken with a 100 x magnification. The scale bar displays a length of 200 µm.*

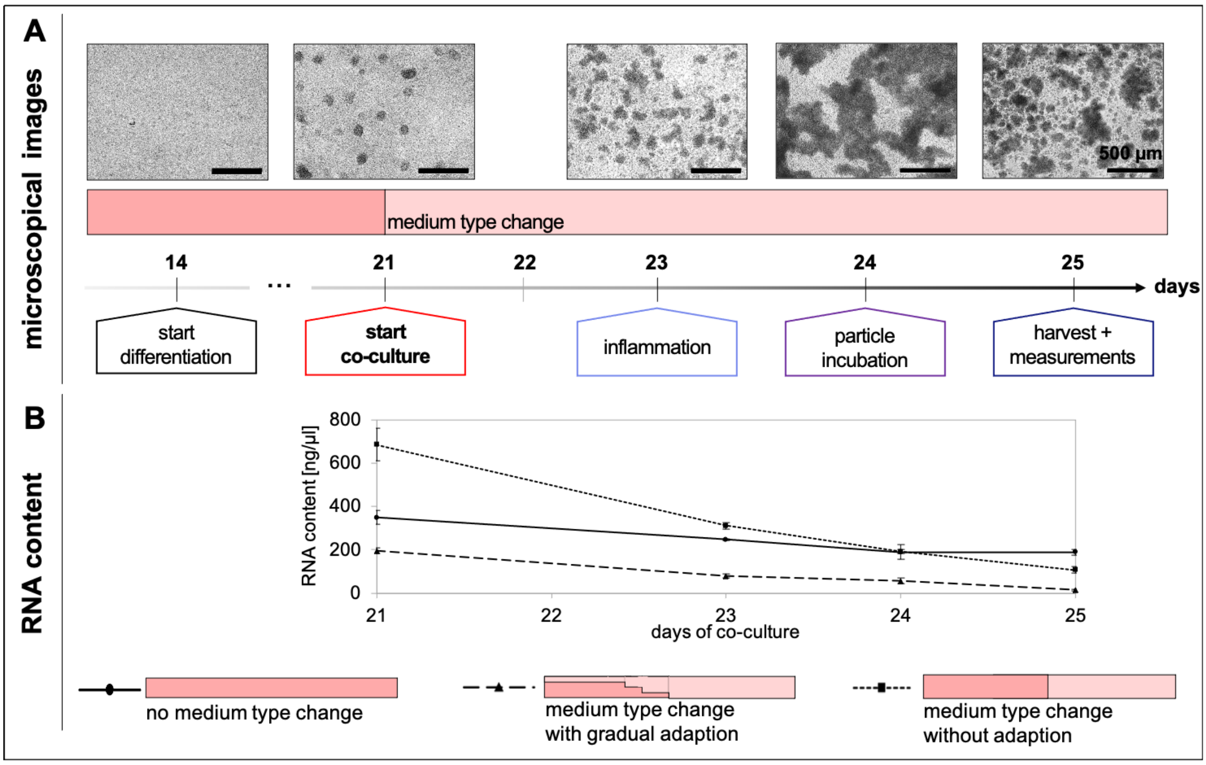


*Figure S 2: Monitoring the survivability of differentiated MUTZ-3 dendritic cells after immediate medium type change from MUTZ-3 to DMEM+ during co-culture simulation procedure.* ***A****: Microscopical examination of MUTZ-3 cell morphology during differentiation in MEM+ and simulation of the co-culture in DMEM+. Images were taken with a 50 x magnification. The scale bar displays a length of 500 µm.* ***B****: Comparison of RNA content of MUTZ-3 cells harvested on days 21, 23, 24 and 25 after being differentiated in MEM+ and receiving 1) no medium type change 2) medium type change with gradual adaption or 3) medium type change without adaption during the co-culture simulation.*

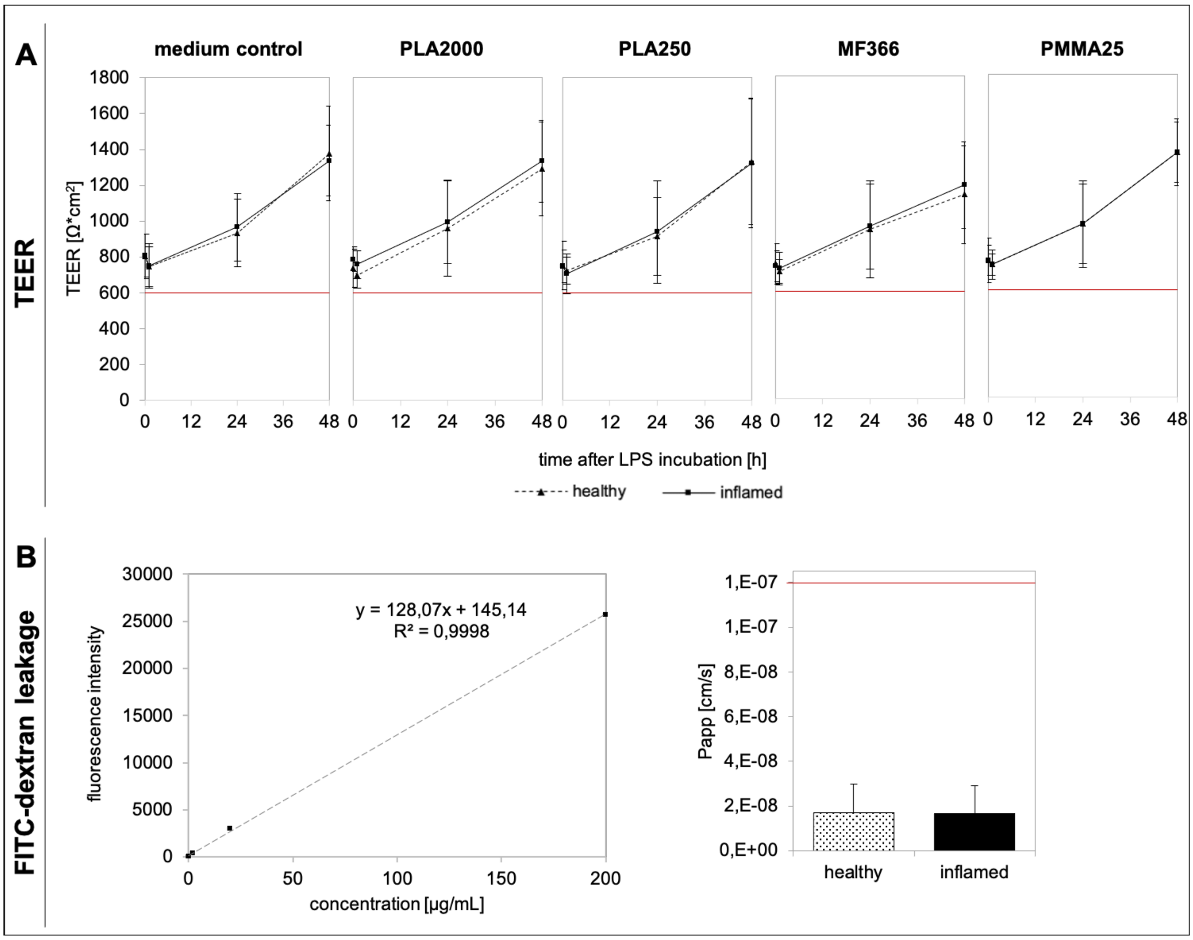


*Figure S 3: Permeability measurements of the co-culture in healthy and inflamed state as well as after incubation with nano-, submicro- and microplastic particles.* ***A:*** *Changes in TEER values after 0 h, 1 h, 24 h and 48 h after LPS incubation. Red lines indicate lower limit for TEER values* ***B****: FITC-dextran leakage in the co-culture model. Left: representative standard curve of FITC-dextran, right: calculated $P_{app}$ values from FITC-dextran leakage after 24 h incubation in the healthy and inflamed model. Red line indicates lower limit for leakage. All data are presented as means ± SD, n = 3.*

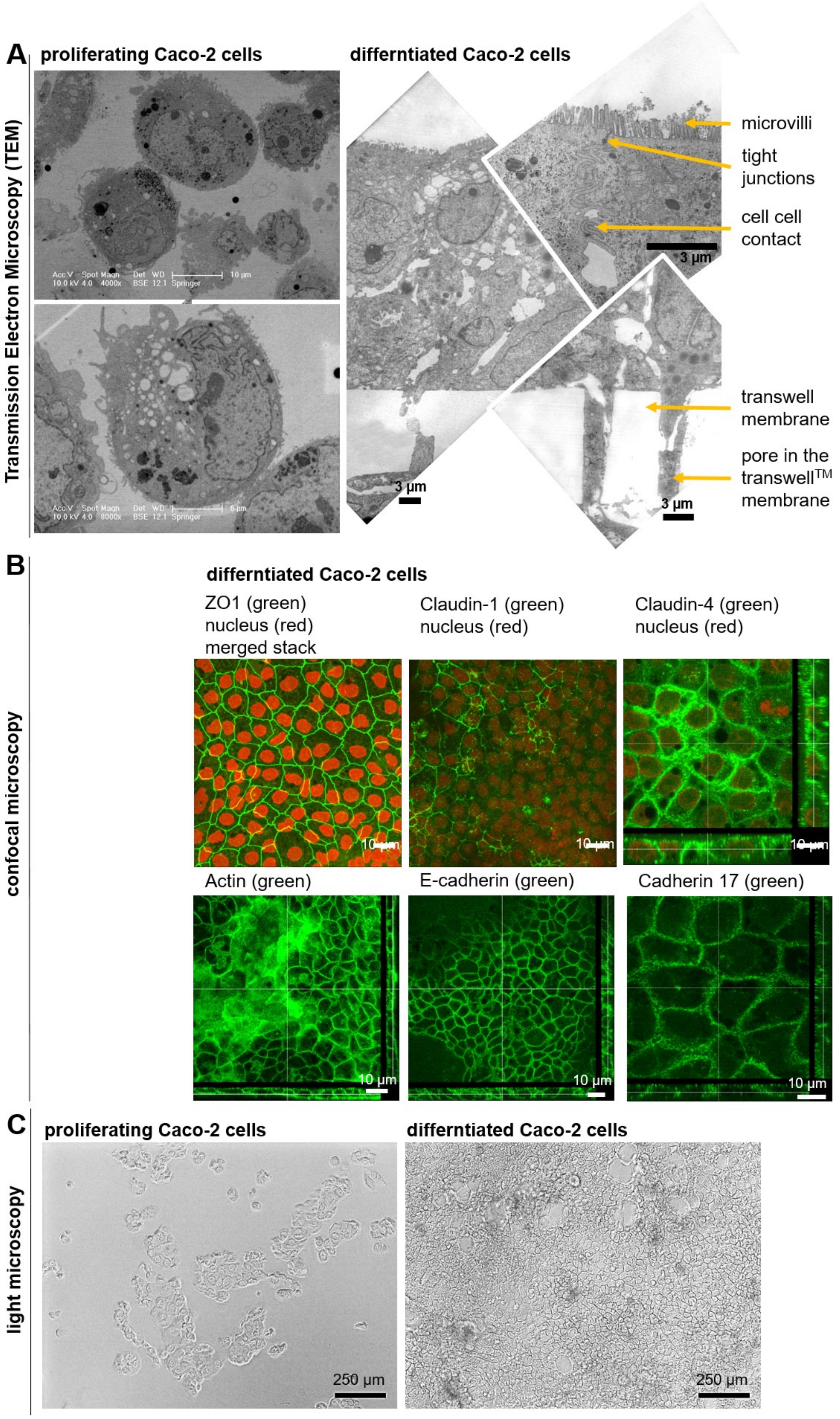
A
Transmission Electron Microscopy (TEM)
proliferating Caco-2 cells
differntiated Caco-2 cells
microvilli
tight junctions
cell cell contact
3 µm
transwell membrane
pore in the transwellTM membrane
3 µm
3 µm
B
confocal microscopy
differntiated Caco-2 cells
ZO1 (green)
nucleus (red)
merged stack
Claudin-1 (green)
nucleus (red)
Claudin-4 (green)
nucleus (red)
10 µm
10 µm
10 µm
Actin (green)
E-cadherin (green)
Cadherin 17 (green)
10 µm
10 µm
10 µm
C
light microscopy
proliferating Caco-2 cells
differntiated Caco-2 cells
250 µm
250 µm

*Figure S 4: Morphological characteristics of differentiated Caco-2 cells.* ***A*** *Transmissions Electron Microscopical images of proliferating and on Transwell™ membrane differentiated Caco-2 cells. Sample preparation: cells were fixed overnight with Karnovsky solution, pre-contrasted in 2% osmium tetroxide, dehydrated, embedded, ultra-thin sections were prepared on the Reichert Ultracuts microtome and then further contrasted with lead and uranyl acetate. Samples were viewed either on a Zeiss 10 CR or an FEI Tecnai G² 20 S-TWIN.* ***B*** *Confocal mikroscopical images of differentiated Caco-2 cells stained with antibodies against tight junction proteins and actin. Sample preparation: Caco-2 cells were differentiated on glass slides, fix, permeabilized and stained for analysis with SP5 from Leica Microsystems.*

Table S1: overview expression markers.

| **cell marker** | **day** | **fold change in gene expression** | | | |
|---|---|---|---|---|---|
| | | **MUTZ-3 without adaption** | **MUTZ-3 with adaption** | **THP-1 without adaption** | **THP-1 with adaption** |
| CD14 | 14 or 18 | 0,9096 | 1,2510 | 1,0300 | 1,0300 |
| | 21 | 0,1078 | 0,0202 | 18,8280 | 18,8280 |
| | 23 | 0,9212 | 0,4855 | 223,9000 | 214,2840 |
| | 24 + | 1,8272 | 1,6281 | 145,7660 | 143,2760 |
| | 25 + | 2,5730 | 0,8373 | 161,7420 | 152,7060 |
| | 24 - | 1,8872 | 0,8142 | 181,1067 | 143,5100 |
| | 25 - | 2,4491 | 1,1078 | 28,7480 | 44,8980 |
| CD1a | 14 or 18 | 1,0183 | 1,0433 | 1,1133 | 1,1133 |
| | 21 | 144,9126 | 14,3687 | 206,6067 | 206,6067 |
| | 23 | 77,0747 | 25,6065 | 153,2433 | 179,0375 |
| | 24 + | 126,6153 | 32,9841 | 46,1975 | 38,0650 |
| | 25 + | 125,8015 | 15,1161 | 32,9775 | 44,9375 |
| | 24 - | 119,3641 | 32,3858 | 9,2875 | 8,4800 |
| | 25 - | 50,3344 | 37,3335 | 0,8725 | 49,5800 |
| CD80 | 14 or 18 | 1,3582 | 0,4460 | 1,0067 | 1,0067 |
| | 21 | 47,6833 | 10,2889 | 0,5800 | 0,5800 |
| | 23 | 46,1654 | 9,6169 | 2,4850 | 5,3475 |
| | 24 + | 27,8826 | 2,7872 | 44,7275 | 95,3875 |
| | 25 + | 27,8574 | 0,8246 | 47,8075 | 29,7025 |
| | 24 - | 45,8369 | 3,0708 | 21,5120 | 10,9750 |
| | 25 - | 31,0697 | 1,3953 | 57,3950 | 160,7900 |
| CD86 | 14 or 18 | 0,8742 | 0,8742 | 1,0250 | 1,0250 |
| | 21 | 4,4245 | 4,4245 | 22,3125 | 22,3125 |
| | 23 | 9,6747 | 9,6747 | 153,6700 | 94,6740 |
| | 24 + | 12,0120 | 8,0763 | 94,2000 | 58,1675 |
| | 25 + | 14,3753 | 9,5050 | 87,2233 | 79,5100 |
| | 24 - | 6,9212 | 5,7715 | 141,1060 | 81,6175 |
| | 25 - | 5,1677 | 3,5524 | 37,6460 | 42,2240 |
| CD209 | 14 or 18 | 1,0231 | 0,6408 | not measured | not measured |
| | 21 | 130,7602 | 26,4726 | | |
| | 23 | 17,3921 | 22,5741 | | |
| | 24 + | 13,1931 | 21,3094 | | |
| | 25 + | 14,3600 | 17,2245 | | |
| | 24 - | 16,3184 | 21,0896 | | |
| | 25 - | 18,5211 | 20,1939 | | |
| IL-10 | 14 or 18 | 0,9702 | 0,7014 | 1,0533 | 1,0533 |
| | 21 | 1,2351 | 0,1935 | 185,9500 | 185,9500 |
| | 23 | 1,2382 | 1,7346 | 423,7280 | 349,8975 |
| | 24 + | 1,6398 | 2,7810 | 1007,4000 | 1450,5067 |
| | 25 + | 2,7299 | 7,6979 | 4087,2525 | 3843,8417 |
| | 24 - | 1,2824 | 1,5027 | 1384,8120 | 1374,9060 |
| | 25 - | 1,8220 | 1,3902 | 1324,8175 | 2285,9475 |

| | | | | | |
|---|---|---|---|---|---|
| IL-8 | 14 or 18 | 1,1350 | 1,3060 | 1,0002 | 1,0002 |
| | 21 | 4,0551 | 1,1251 | 5,1600 | 4,2067 |
| | 23 | 3,6889 | 1,7545 | 3,1550 | 3,7620 |
| | 24 + | 2,3910 | 2,3703 | 13,5550 | 43,3625 |
| | 25 + | 2,8888 | 2,9049 | 7,4017 | 20,0080 |
| | 24 - | 1,6176 | 1,3302 | 4,6740 | 3,5550 |
| | 25 - | 1,6747 | 1,6252 | 0,0740 | 0,1917 |
| IL-6 | 14 or 18 | 0,7827 | 0,7827 | 1,0000 | 1,0000 |
| | 21 | 1,8403 | 1,8403 | 7,1700 | 7,1700 |
| | 23 | 1,4096 | 1,4096 | 2,0960 | 3,2180 |
| | 24 + | 2,2503 | 1,8523 | 8,4040 | 7,3500 |
| | 25 + | 2,4457 | 0,7056 | 6,4560 | 10,3900 |
| | 24 - | 1,8445 | 1,9969 | 5,7300 | 2,2680 |
| | 25 - | 1,6933 | 1,3645 | 5,1600 | 4,8317 |
| TNF-α | 14 or 18 | 0,9579 | 0,6231 | 1,0533 | 1,0533 |
| | 21 | 70,8333 | 11,3959 | 0,7380 | 0,7380 |
| | 23 | 37,4491 | 25,5557 | 9,1580 | 2,2160 |
| | 24 + | 22,0109 | 17,8806 | 0,8680 | 0,9983 |
| | 25 + | 33,3960 | 11,1376 | 1,9620 | 1,1540 |
| | 24 - | 24,3259 | 12,9052 | 1,1900 | 1,1825 |
| | 25 - | 55,7913 | 23,0738 | 0,9583 | 0,8760 |

Legend:

| | |
|---|---|
| 14 or 18 | start of differentiation |
| 21 | start of co-cultivation |
| 23 | medium change or inflammation with LPS |
| 24 + | medium change, 24h after LPS inflammation |
| 25 + | medium change, 48h after LPS inflammation, potential incubation with substances |
| 24 - | medium change only |
| 25 - | medium change only, potential incubation with substances |
